# Domestic dog demographics and estimates of canine vaccination coverage in a rural area of Zambia for the elimination of rabies

**DOI:** 10.1371/journal.pntd.0009222

**Published:** 2021-04-28

**Authors:** Chiho Kaneko, Ryosuke Omori, Michihito Sasaki, Chikako Kataoka-Nakamura, Edgar Simulundu, Walter Muleya, Ladslav Moonga, Joseph Ndebe, Bernard M. Hang’ombe, George Dautu, Yongjin Qiu, Ryo Nakao, Masahiro Kajihara, Akina Mori-Kajihara, Herman M. Chambaro, Hideaki Higashi, Chihiro Sugimoto, Hirofumi Sawa, Aaron S. Mweene, Ayato Takada, Norikazu Isoda

**Affiliations:** 1 Unit of Risk Analysis and Management, Hokkaido University Research Center for Zoonosis Control, Sapporo, Hokkaido, Japan; 2 Division of Bioinformatics, Hokkaido University Research Center for Zoonosis Control, Sapporo, Hokkaido, Japan; 3 Division of Molecular Pathobiology, Hokkaido University Research Center for Zoonosis Control, Sapporo, Hokkaido, Japan; 4 Department of Disease Control, School of Veterinary Medicine, The University of Zambia, Lusaka, Zambia; 5 Macha Research Trust, Choma, Zambia; 6 Department of Biomedical Sciences, School of Veterinary Medicine, The University of Zambia, Lusaka, Zambia; 7 Department of Para-Clinical Studies, School of Veterinary Medicine, The University of Zambia, Lusaka, Zambia; 8 Virology Unit, Central Veterinary Research Institute, Lusaka, Zambia; 9 Ministry of Fisheries and Livestock, Lusaka, Zambia; 10 Hokudai Center for Zoonosis Control in Zambia, Hokkaido University Research Center for Zoonosis Control, Sapporo, Hokkaido, Japan; 11 Laboratory of Parasitology, Faculty of Veterinary Medicine, Graduate School of Infectious Diseases, Hokkaido University, Sapporo, Hokkaido, Japan; 12 Division of Global Epidemiology, Hokkaido University Research Center for Zoonosis Control, Sapporo, Hokkaido, Japan; 13 Division of Infection and Immunity, Hokkaido University Research Center for Zoonosis Control, Sapporo, Hokkaido, Japan; 14 Division of Collaboration and Education, Hokkaido University Research Center for Zoonosis Control, Sapporo, Hokkaido, Japan; University of Surrey, UNITED KINGDOM

## Abstract

**Background:**

An estimated 75% or more of the human rabies cases in Africa occur in rural settings, which underscores the importance of rabies control in these areas. Understanding dog demographics can help design strategies for rabies control and plan and conduct canine mass vaccination campaigns effectively in African countries.

**Methodology/Principal findings:**

A cross-sectional survey was conducted to investigate domestic dog demographics in Kalambabakali, in the rural Mazabuka District of Zambia. The population of ownerless dogs and the total achievable vaccination coverage among the total dog population was estimated using the capture-recapture-based Bayesian model by conducting a canine mass vaccination campaign. This study revealed that 29% of the domestic dog population was under one year old, and 57.7% of those were under three months old and thus were not eligible for the canine rabies vaccination in Zambia. The population growth was estimated at 15% per annum based on the cross-sectional household survey. The population of ownerless dogs was estimated to be small, with an ownerless-to-owned-dog ratio of 0.01–0.06 in the target zones. The achieved overall vaccination coverage from the first mass vaccination was estimated 19.8–51.6%. This low coverage was principally attributed to the owners’ lack of information, unavailability, and dog-handling difficulties. The follow-up mass vaccination campaign achieved an overall coverage of 54.8–76.2%.

**Conclusions/Significance:**

This paper indicates the potential for controlling canine rabies through mass vaccination in rural Zambia. Rabies education and responsible dog ownership are required to achieve high and sustainable vaccination coverage. Our findings also propose including puppies below three months old in the target population for rabies vaccination and emphasize that securing an annual enforcement of canine mass vaccination that reaches 70% coverage in the dog population is necessary to maintain protective herd immunity.

## Author summary

Because dogs are the main transmitter of rabies to humans, controlling rabies in dogs is essential for preventing rabies in humans. Canine vaccination is well-known as the most effective measure for controlling rabies in dogs. Understanding the demographics and dynamics of dog populations is important when designing efficient canine vaccination strategies. Furthermore, protective herd immunity in the total dog population should be attained through the vaccination of owned dogs since ownerless dogs are not usually covered in such campaigns. This study investigated domestic dog demographics and estimated the number of ownerless dogs to finally estimate the vaccination coverage among the overall dog population achievable through a mass vaccination campaign in a rural setting in Mazabuka District, Zambia. The target domestic dog population was young, and population growth was estimated at 15% annually based on the cross-sectional survey. The vaccination coverage attained by providing free canine mass vaccination campaigns was eventually estimated as 54.8–76.2% in the overall dog population, coupled with the estimate that the ownerless dog population was quite small. Our findings emphasize the necessity of conducting annual canine mass vaccinations, including puppies, that target 70% coverage in the dog population to maintain protective herd immunity.

## Introduction

Rabies is one of the most feared, fatal zoonotic diseases in the world; it causes approximately 59,000 human deaths worldwide each year, with over 95% of cases occurring in Asian and African countries [1]. Although rabies may affect all species of warm-blooded animals, the large majority of human rabies cases are intermediated by dogs in Asia and Africa [2]. Therefore, in addition to providing human post-exposure prophylaxis (PEP), canine vaccination is a key measure to control dog-mediated human rabies [3–6]. Despite the presence of established control measures, rabies remains endemic in over 100 countries and territories [4] because of the low public awareness of rabies, low prioritization of rabies control, poor registration of owned dogs, insufficient management of stray dogs, unavailability of high-quality animal vaccines, lack of resources required to implement control programs, and the presence of wild animals that share rabies infections. Furthermore, particularly in rural communities, the low availability of rapid and appropriate PEP makes controlling human rabies cases difficult [3]. Based on the estimate that over 75% of human rabies cases in Africa occur in rural settings [7], it is thus important to establish sustainable and suitable control measures in rural settings in an effort to effectively control rabies.

To prevent a rabies outbreak in a dog population, 20–45% of the dog population must always be immune; this threshold is recognized as the critical vaccination coverage of rabies [8]. This is calculated from the basic reproductive number of rabies, which is estimated to be between 1 and 2 around the world [8]. Canine mass vaccination campaigns are commonly implemented to immunize dogs in rabies endemic countries, particularly in Asia and Africa. To maintain herd immunity beyond the aforementioned critical threshold coverage in the interval between vaccination campaigns, a higher vaccination coverage must be achieved in the dog population during those campaigns [8]. This high coverage must be achieved because of the rapid decline in herd immunity due to the death of immunized dogs, the birth and immigration of susceptible dogs [9], and the loss of individual immunity [10]. Therefore, the actual vaccination coverage that should be achieved during one campaign depends on the dog population dynamics, the duration of vaccine-induced immunity, and the interval between campaigns. Empirically, a vaccination coverage of at least 70% of the dog population has been recognized as the coverage required in mass vaccination campaigns that are generally conducted annually [4,11]. Recently, this empirically derived consensus was verified by a study that used retrospectively collected dog demographic data in Tanzania [8] and in studies that used prospectively collected cohort data in South Africa and Indonesia [9,12]. These studies estimated that a target vaccination coverage of 60–70% is sufficient to avoid coverage falling below the critical threshold of 20–45% in those studied dog populations through annual mass vaccination campaigns. However, the empirically observed levels of coverage that have successfully controlled rabies vary according to the circumstances [13,14]. Vaccination campaigns that do not reach 70% of the dog population can sometimes be effective, but they often fail to prevent rabies outbreaks, which are primarily affected by the dog demographic characteristics (such as rapid turnover) of each population that contribute to the decline of coverage [8]. Thus, understanding the dynamics and demographics of dog populations in rabies endemic countries can help design strategies for controlling rabies in dogs and humans and plan and conduct canine mass vaccination campaigns effectively to utilize limited resources.

Rabies is considered endemic in all regions of the Republic of Zambia that annually report several clinically diagnosed human cases, several dozen more cases in animals (suspected and diagnosed), and several hundred to thousands of dog bite cases [15–17]. Moreover, the costs of PEP and rabies immunoglobulin disbursement coupled with mortality place a huge burden on the public health sector [16]. However, rabies reporting in Zambia has been inconsistent, with various studies reporting different figures [15,16,18]; this is possibly attributed to poor surveillance and a lack of collaboration and communication between human and animal health sectors [19]. Hence, considering common situations in rabies endemic countries (such as inadequate laboratory and transport infrastructure in addition to the aforementioned situations [20,21]), the number of rabies cases reported in Zambia is very likely underestimated, and the actual disease burden of rabies could be much higher.

Rabies control in Zambia through canine vaccination has been implemented similarly to that in other African countries. The dog population in Zambia was reported to be 483,628 between 2004 and 2009, as estimated by the National Livestock Epidemiology and Information Center of Zambia [22]. In Zambia, dogs are sometimes confined to their houses or premises, which are surrounded by fences and block walls in urban settings, such as the capital city of Lusaka; conversely, in rural areas, dogs are mostly allowed to freely roam and kept without confinement using chains or collars. Although several reports on the rabies situation in Zambia have been published [15,22–25], only a few papers have discussed vaccination coverage in dog populations [26,27]. De Balogh et al. reported the vaccination status among dogs in an urban area and a semi-rural area and the vaccination coverage attained through central-point mass vaccination in the urban area [26]. Mulipukwa et al. reported the vaccination status among dogs in Nyimba District, which is a rural district located in Eastern Province [27]. However, no information regarding vaccination coverage attainable through central-point mass vaccination in the rural areas of Zambia is available. A substantial number of mass vaccination campaigns in dogs have been conducted in Zambia, but the attained coverage has been poorly investigated and assessed, including the factors that likely influence the success of campaigns (e.g., owners’ willingness to pay for vaccination, household density, or campaign styles matched to lifestyle and land-use, etc. [6,28,29]). Similarly, no previous studies have reported on the dog demographics or dynamics in rural settings of Zambia even though such information is crucial for designing and planning effective vaccination strategies matched to the target dog community.

Hence, this study aimed to elucidate dog demographics and assess the vaccination coverage achievable through a canine mass vaccination campaign in rural settings of Zambia with an eventual goal of verifying the feasibility of eliminating rabies from dogs in Zambia. To attain the above aims, this study involved 1) estimating the ownerless dog populations, 2) investigating the demographics of the domestic dog population, 3) conducting mass vaccination campaigns (the first mass vaccination and the follow-up mass vaccination), 4) estimating the vaccination coverage attained through the campaigns, and 5) revealing the owners’ knowledge, attitude, and practices for rabies and its control, which influences achievable vaccination coverage, in a rural setting of Zambia.

## Methods

### Ethical approval

Ethical approval for this study was obtained from the Ministry of Fisheries and Livestock of the Government of the Republic of Zambia. This study was conducted under the monitoring project of the Ministry of Fisheries and Livestock, therefore, this study was not categorized as animal experiments. For human participant, verbal formal consent was obtained from each participant.

### Study area

**Fig 1:**
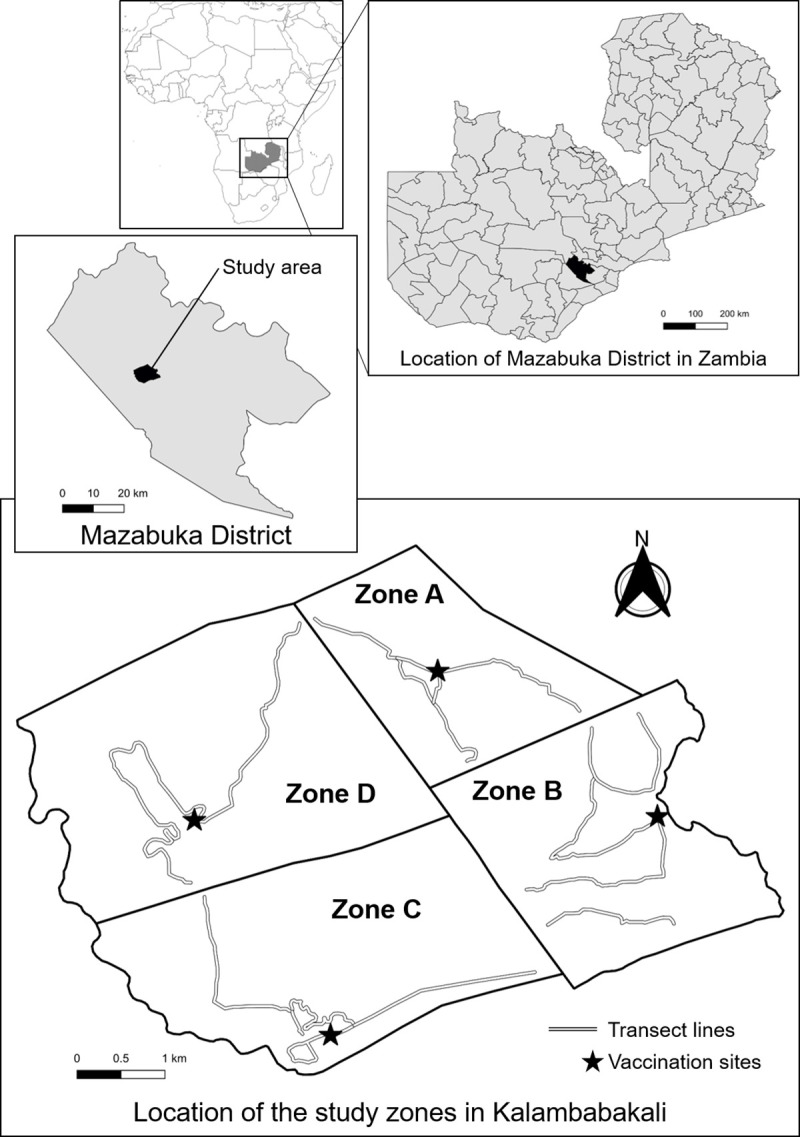
Location of the study area in Kalambabakali, Mazabuka District of Zambia. Study area consists of four continuous zones (Zone A, B, C and D) in Kalambabakali. Map of the African Continent was obtained from the Natural Earth (https://www.naturalearthdata.com/). Map of Zambia was downloaded from the Humanitarian Data Exchange (https://data.humdata.org/dataset/zambia-administrative-boundaries-level-1-provinces-and-level-2-districts-with-census-2010-population), which is shared under Creative Commons Attribution for Intergovernmental Organizations license (https://creativecommons.org/licenses/by/3.0/igo/legalcode). The shapefiles provided under this license themselves were not modified, but the shapefiles originally created for representing study area were overlaid on the shapefiles corresponding to Mazabuka District. Maps were created using the QGIS 3.10 software (https://qgis.org/en/site/).

Two canine mass rabies vaccination campaigns and a survey on canine demographic characteristics and vaccination coverage estimates were conducted in Kalambabakali in Mazabuka District of Zambia. Mazabuka District (15.86°S, 27.76°E), which is located in Southern Province, has a total human population of 230,972 individuals (2010 census), approximately 13,000–14,000 dogs (in 2010–2013; according to Mazabuka district veterinary office [DVO] reports), and an area of 6,242 km^2^ [30] (A part of Mazabuka District has been separated and is known as Chikankata District in 2011). Several hundred dog bite cases are recorded in this region annually, with a total of 61–360 cases reported annually between 2010 and 2015 (Annual Reports, Department of Veterinary Services). In 2014, one canine and one bovine suspected rabies case tested positive, and an additional 147 suspected canine cases were reported (Annual Reports, Department of Veterinary Services). The study area consists of four continuous zones. Zones A and D correspond to the administratively subdivided areas of village 2 and the Mukuyu area, respectively, in Kalambabakali. Zones B and C correspond to village 3 and village 4, respectively, in the same region (Fig 1). All zones were well defined by administrative boundaries. The total area of the study zones was approximately 30.6 km^2^. Zones A, B, C, and D have areas of 4.6, 7.0, 8.0, and 11.0 km^2^, respectively. These zones are located in rural areas, approximately 17 km from central Mazabuka. The main agricultural activities in this region include maize and cotton cultivation and livestock rearing.

### The first canine mass vaccination (capture)

Two mass vaccination campaigns were conducted in this study: the first mass vaccination campaign (capture) and the follow-up mass vaccination (described in the latter section). The first mass vaccination campaign was subsequently followed by the transect survey and household survey while the follow-up mass vaccination campaign was conducted three weeks after the first mass vaccination campaign. Mazabuka DVO staff distributed posters announcing the vaccination campaign a week prior in the target zones. The posters were written in both English and the local language and displayed in front of schools, clinics, and houses belonging to the local chiefs, where people commonly gather. Additionally, Mazabuka DVO staff traveled by motorcycles in the target zones to publicly use loudspeakers to advertise the upcoming campaign several times during the week before the vaccination campaign.

A central-point canine vaccination campaign was conducted from 9:00 to 13:00 in zone B and from 14:00 to 18:00 in zone A on May 21, 2016 (Saturday). Vaccinations were held from 9:00 to 13:00 in zone D and from 14:00 to 18:00 in zone C on May 22, 2016 (Sunday). Each zone had one vaccination spot: the dip tank site in zone A, the school in zone B and D, and the local chief’s premise in zone C. Four veterinary assistant officers from the DVO administered the vaccines and issued the vaccination certificates. One local livestock officer was also present during the campaign. Human PEP anti-rabies vaccines (Verorab; Sanofi Pasteur, Lyon, France) and disinfectants were also provided in case of any dog bites. The dogs were vaccinated subcutaneously with 2 ml of Rabies Alum Adjuvant Vaccine (Central Veterinary Research Institute, Lusaka, Zambia) using a single syringe and needle for each animal. The vaccine used was a locally produced rabies vaccine that is commonly provided and used by the DVO in the target zones. The vaccines were distributed free of charge. Dogs aged less than three months and those that were obviously unhealthy were not vaccinated as per the rabies vaccination guidelines of the vaccine manufacturer and the “Rabies Disease Control in Zambia Protocol” based on the Control of Dogs Act, Cap 247 of the Laws of Zambia. A strict cold chain was observed, and the vaccinated dogs were labeled with color spray on their bodies and issued a Government of the Republic of Zambia rabies vaccination certificate. Additionally, information about the owners’ names and addresses, and the dogs’ names, age, sex, color, markings, and vaccination history were recorded.

### Transect survey (the first recapture)

Vaccination coverage was assessed in the target zones using the capture-recapture method described earlier by Kayali et al. [31]. Two transect teams comprising four observers who each counted all dogs encountered in the transect lines were organized. Dogs labeled with the color spray (vaccinated) were distinguished from unlabeled dogs (unvaccinated). The two teams used cars traveling at 15 to 20 km/h for the transect survey to avoid accidental bite injuries. The transect survey in each zone was conducted in the morning on the first day following the mass vaccination campaign (May 23, 2016). We conducted one transect survey in each zone, although this survey should ideally be conducted several times per area to avoid biased observations. Only one survey was performed because of the difficulty to adjust the schedule of the DVO staff due to other administrative affairs. The main roads were selected as the transect lines in each target zone. Additionally, a 50-m wide buffer around the boundary of each zone was established to avoid counting migrating (even temporarily) dogs from outside the zone in our survey. During the transect survey, we carried Global Positioning System (GPS) tracking devices to record log of our movement. The length of the transect lines were measured via Google Earth Pro software (2015 Google) with the record of the GPS log. The total length of the transect lines in each zone were as follows: 5.12 km in zone A, 8.91 km in zone B, 7.80 km in zone C, and 8.08 km in zone D.

### Household survey (the second recapture)

Household surveys were conducted on day 5 after the mass vaccination campaign and were continued for another five days. The household survey targeted all households in the study area regardless of whether they owned dogs for assessing the number of owned dogs and humans and owned dog demographic characteristics. Each household in the study area was visited during this time, and the heads of the households were interviewed. If the head of the household was absent, a suitable substitute was chosen for the interview. All respondents were told the purpose of the study, and their consents for participation were obtained. The questionnaire (S1 Appendix) was written in English, but the interview was performed in the local language if necessary. Information on the number of dogs in the household and the presence of spray-marks on any dogs were collected, as were each dog’s age and sex. Each dog’s previous vaccination history and its validity were also assessed. Dog owners were asked to provide their reasons for not participating in the first mass vaccination campaign, when applicable. The owners were also asked about their knowledge of rabies to assess whether they had accurate information on rabies. Furthermore, they were asked about the affordability of the canine rabies vaccination (willingness to pay) and what they had actually paid for the vaccination (actual cost). The confinement probability was estimated by confirming whether each dog was confined to each household’s premises (e.g., by chain or cage).

### Follow-up mass vaccination

A follow-up mass vaccination campaign was provided three weeks after the first mass vaccination campaign for owners who missed the first campaign. The follow-up campaign was held at the same locations as the original campaign. Flyers were distributed to each household during the household survey described above to advertise the follow-up campaign. The other conditions of the follow-up mass vaccination campaign were the same as those in the first campaign.

### Data analysis to estimate the ownerless dog population and total vaccination coverage

The Bayesian model modified from Kayali et al. was used in this study [31]. In each study zone *i* (*i* = 1, 2, 3, and 4 corresponding to zones A, B, C, and D, respectively), all vaccinated dogs were labeled with color spray during the mass vaccination campaign.

We modelled the sampling process of the capture-recapture study. First, we defined an owned dog as a dog kept by a human and belonging to a household. We also defined an ownerless dog as a dog that is not kept by a human and does not belong to a household. During the transect survey, dogs were distinguished by whether they were marked or unmarked. Since there was no way to determine whether an unmarked dog was owned, the number of unmarked dogs observed in study zone *i*, *Z*_*i*_, can be written as follows:

$$Z_{i}=X_{2,i}+Y_{i},$$ (1)

in which *X*_2,*i*_ and *Y*_*i*_ denote the number of unmarked dogs that were owned and the number of unmarked dogs that were ownerless and were recaptured during the transect survey in a given zone *i*, respectively. All of the marked dogs were owned dogs since ownerless dogs were not brought to the mass vaccination campaign. *X*_1,*i*_ represents the number of marked, owned dogs that were recaptured during the transect survey in a given zone *i*.

The recapture process in our capture-recapture survey was assumed to follow a binomial sampling process with a recapture probability that is equal among all dogs (marked owned, unmarked owned, and ownerless) but differed by zone. Hereafter, we refer to the recapture probability in zone *i* as *p*_*i*_. The probability of the number of marked and unmarked dogs recaptured in a given study zone *i*, *X*_1,*i*_ and *Z*_*i*_, can be written as follows:

$$X_{1,i}∼Bin\left((1-c_{1,i})M_{v,i},p_{i}\right),$$ (2)

$$Z_{i}∼Bin\left((1-c_{2,i})M_{u,i}+N_{i},p_{i}\right),$$ (3)

in which *Bin* denotes binomial distribution, *c*_1,*i*_ and *c*_2,*i*_ are confinement probabilities related to zone *i* for owned marked and owned unmarked dogs, respectively; *M*_*u*,*i*_ is the total number of unvaccinated owned dogs; and *N*_*i*_ is the total number of ownerless dogs in zone *i*. The total number of vaccinated (marked and owned) dogs in zone *i*, *M*_*v*,*i*_, was obtained from the registration at the vaccination point. A description of each parameter is listed in Table 1.

**Table 1:** Model parameters.

Parameter	Description	Source
*M*_*i*_	The total number of owned dogs in zone *i*	estimated
*M*_*v*,*i*_	The total number of vaccinated (marked and owned) dogs during the mass vaccination in zone *i*. This was obtained from the registration at the vaccination point	observed
*m*_*i*_	Number of recaptured marked (vaccinated) dogs in the household survey in zone *i*	observed
*n*_*i*_	Number of recaptured dogs in the household survey in zone *i*	observed
*N*_*i*_	Total number of ownerless dogs in zone *i*	estimated
*a*_*i*_	Ratio of ownerless dogs to owned dogs in zone *i*, written as *N*_*i*_ = *a*_i_*M_*i*_	estimated
*p*_*i*_	Recapture probability, written as *p*_*i*_ = *C*_i_*E_i_*R_*i*_	estimated
*C*_*i*_	Coverage stands for the area covered by the transect line	observed
*E*_*i*_	Probability of encountering a specific dog given the area	observed
*R*_*i*_	Recording probability of the observer actually recording an encountered dog	observed
*c*_1,*i*_	Confinement probability for owned marked dogs	estimated
*c*_2,*i*_	Confinement probability for owned unmarked dogs	estimated
*X*_1,*i*_	Number of marked dogs observed during the transect survey in zone *i*	observed
*Z*_*i*_	Number of unmarked dogs observed during the transect survey in zone *i*	observed

The model parameters with Bayesian inference were estimated using the Markov chain Monte Carlo simulations in the OpenBUGS software (version 3.2.3 rev 1012).

Likelihood was determined as the product of probability mass functions for the observed data of the marked and unmarked dogs during the transect survey as follows:

$$Likelihood=pmf(X_{1,i},X_{1,i}^{obs})pmf(Z_{i},Z_{i}^{obs}),$$ (4)

in which pmf(*x*,*y*) denotes the probability mass function describing the probability of observing *y* with a distribution *x*.

The total number of owned dogs in each study zone was initially estimated using the Chapman estimate formula [32–34] via data collected from the household survey:

$$M_{i}=\left[\frac{\left(M_{v,i}+1)(n_{i}+1\right)}{\left(m_{i}+1\right)}\right]-1$$ (5)

and variance:

$$var(M_{i})=\frac{\left(M_{v,i}+1)(n_{i}+1)(M_{v,i}-m_{i})(n_{i}-m_{i}\right)}{{\left(m_{i}+1\right)}^{2}(m_{i}+2)},$$ (6)

in which *n*_*i*_ and *m*_*i*_ are the numbers of recaptured dogs and recaptured marked (vaccinated) dogs in the household survey in zone *i*, respectively. These estimates specify the parameters of a normal prior distribution that was adopted for *M*_*i*_. The other prior distributions were also obtained from data collected during the household survey. More information can be found in the supplementary material (S2 and S3 Appendix and S1 Table). Vaccination coverage was calculated as the proportion of actual vaccinated dogs during each of the first and follow-up mass vaccination campaigns in the owned and overall dog populations estimated via Bayesian modeling.

### Dog demographics and projection of dog population growth

A static life table and a female fecundity table [35,36] were constructed based on dog information collected during the household survey. The collected information included: i) the number of dogs currently owned, ii) the sex and age of all dogs, and iii) the reproductive history of female dogs (the number of litters in a lifetime and within the last 12 months and the size of the most recent litter). Static life tables can be calculated directly from a stationary age distribution only when the frequency of each age class *x* is equal to or greater than that of *x* + 1 [35]. To construct a static life table, the observed dog frequency in each age class was smoothed by fitting the data of the age distribution of dogs with a statistical model describing age structure [35] as follows:

$$\log\left(n_{a}\right)=\alpha +\beta a+\gamma a^{2},$$ (7)

in which, *n*_*a*_ denotes the number of dogs aged *a*. The parameters *α*, *β*, and *γ* were estimated by a nonlinear least squares regression with the model as above. By substituting estimated values of *α*, *β*, and *γ*, we obtained the smoothed number of dogs per age and completed the static life table. The data on age and sex of 861 of 872 dogs was converted into a static life table after excluding the data of 11 dogs whose age was unidentified (S2 Table). The information obtained from 334 female dogs, excluding females whose fecundity data were not complete among the total females (*n* = 374), was used for constructing a female fecundity table (S3 Table). The formulas used to construct the static life table and female fecundity table are provided in S4 Appendix. The population growth was projected by means of an age-structured, population projection matrix (Leslie matrix) [36], under the assumption that the environment remained constant and no emigration or immigration occurred in the dog population. The impact of survival and fecundity in different age classes was assessed via an elasticity analysis. These analyses relating to the Leslie matrix were performed using the R package “demogR” in R 3.6.3 [37].

### Statistical analysis

According to the dog owners’ willingness to pay for a vaccine and what they had actually paid for a single canine rabies vaccination, we constructed reverse cumulative vaccination probability curves based on the vaccination cost to evaluate the expected vaccination coverage, which relies on owners’ willingness. A log-rank test was performed on the curves of willingness to pay and what owners actually paid to compare the expected decrease in vaccination coverage as the cost increases using the R package “survival” in R 3.6.3 [37]. A *p* value of < 0.05 was considered statistically significant.

## Results

### Household and dog population characteristics

During the household survey, we visited 333 households that owned at least one dog and 177 households that did not own any dogs. In total, 510 households were visited (Table 2). In the study area, 3.6% of households were missed because the residents were absent or simply because the house owners refused to participate in the survey. A total of 3,882 people were covered by the survey, and the mean number of persons per household was 7.6 (8.6 among the dog-owning household group), except for two households whose data were unavailable. In total, 872 of the owned dogs were covered in the household survey. The characteristics of the dog population are exhibited in Table 3. A total of 29% of dogs in the study area were young dogs (under one year old) based on the information from the household survey. Of these dogs, 57.7% were less than three months old and thus were ineligible for vaccination according to the vaccine manufacturer and the “Rabies Disease Control in Zambia Protocol.” The owners reported various reasons for owning their dogs (*n* = 333; because of multiple answers, a total of 379 answers were reported including four unavailable answers) such as for guarding (98.2%), hunting (13.5%), as a pet (0.6%), and for breeding (0.3%).

**Table 2:** Number of households involved in the study.

	Zone A	Zone B	Zone C	Zone D	Total in the study area
Total number of households	89	176	100	145	510
Number of dog-owning households among total number of households	66	115	51	101	333

**Table 3:** Characteristics of the studied dog population.

Total number of dogs involved in the survey		872
Human-to-dog ratio		4.45:1
Male-to-female ratio in dogs (except for 15 dogs whose sex was not identified)		1.27:1
Number of dogs in a dog-owning household	Mean	2.6
	Median	2
Age (except for 11 dogs whose age was not identified)	Mean (years old)	2.7
	Median (years old)	2

### Demographics and population growth in the owned dog population

Age-specific mortality was highest in the dogs under one year old (47%) according to the static life table (S2 Table). The life expectancy at birth was 3.17 years. The sex-specific static life tables indicated tendencies showing that the age-specific survival (particularly in the reproductive age class) and the age-specific life expectancy in female dogs were lower than those in male dogs (S4 Table). Females began breeding aged 0.75 years as observed in the survey. Their reproductive period continued up to the age of 14 years on the basis of owners’ reports. The mean litter size was 4.3 puppies (95% confidence interval: 4.0–4.6). Female fecundity is summarized in S3 Table.

The dog population growth projection from the Leslie matrix is described as follows. Population growth (*λ*) was estimated at 1.15. The net reproductive rate (*R*_0_), which is defined as the mean number of female offspring that a female produces during her lifetime, was 1.93. The generation time, which is defined as the mean parental age at which all offspring are born, was estimated at 4.6 years. The intrinsic growth rate (*r*), which is a measure of the instantaneous rate of change of population size per individual, was 0.14. An elasticity analysis of the Leslie matrix identified the survival of dogs under one year old to have the greatest proportional effect on the change of the dominant eigenvalue *λ*, accounting for 0.23 of the elasticity. Survival of the age class 1–2 (e = 0.20), followed by survival of the age classes 2–3 (e = 0.13) and 3–4 (e = 0.09) also influenced population growth.

### Ownerless dog population and vaccination coverage estimates

A total of 392 dogs were vaccinated at the four vaccination points during the first mass vaccination campaign in the study zones (74 in zone A, 146 in zone B, 74 in zone C, and 69 in zone D, including 29 dogs from outside the zones). Three hundred dogs were vaccinated in the follow-up mass vaccination campaign (55 in zone A, 89 in zone B, 9 in zone C, and 122 in zone D, including 25 dogs from outside the zones). The median ownerless dog population was estimated at 11 (95% credible interval [CI]: 0–40) in zone A, 5 (95% CI: 0–29) in zone B, 2 (95% CI: 0–10) in zone C, and 15 (95% CI: 0–76) in zone D. The ratio of ownerless to owned dogs was 0.06 (95% CI: 0.00–0.23), 0.02 (95% CI: 0.00–0.10), 0.01 (95% CI: 0.00–0.08), and 0.05 (95% CI: 0.00–0.23) in zones A, B, C, and D, respectively. Vaccination coverage in the owned dog population attained through the first mass vaccination campaign was estimated at 20.9–52.6% in the four zones and was almost similar to the coverage among the overall dog population because there were so few ownerless dogs (Table 4). Vaccination coverage attained through the follow-up mass vaccination campaign was increased to 57.9–77.8% in owned dogs, but it did not reach the 70% coverage recommended by the World Health Organization (WHO) in zones C and D (Table 5). S5 Table shows the posterior distributions and S6 Table shows the summary of the sensitivity analysis. S7 Table shows the proportion of vaccinated owned dogs based on the observation in the household survey.

**Table 4:** Estimated vaccination coverage in owned and overall dog populations through the first mass vaccination campaign.

Zone A	Zone B	Zone C	Zone D
Vaccination coverage in the owned dog population (%)			
41.3	48.3	52.6	20.9
(38.9–44.1)	(46.9–49.8)	(50.0–55.4)	(19.3–22.8)
Overall vaccination coverage (%)			
38.7	47.3	51.6	19.8
(33.7–42.3)	(43.9–49.2)	(48.2–54.7)	(16.8–22.1)

**Table 5:** Estimated vaccination coverage in owned and overall dog populations through the follow-up mass vaccination campaign.

Zone A	Zone B	Zone C	Zone D
Vaccination coverage in the owned dog population (%)			
72.0	77.8	59.0	57.9
(67.8–76.8)	(75.5–80.2)	(56.1–62.2)	(53.5–63.1)
Overall vaccination coverage (%)			
67.4	76.2	57.9	54.8
(58.7–73.7)	(70.6–79.3)	(54.0–61.3)	(46.4–61.1)

Values in parentheses are 95% credible intervals

### Reasons for non-participation in the first mass vaccination

A total of 152 owners participated in the first mass vaccination campaign out of the 333 dog-owning households visited during the household survey. The owners who did not participate in the first mass vaccination campaign were asked why they did not participate (S8 Table). The most common reason was that the owner had not been informed about the mass vaccination beforehand (32.0%). The second and third most common reasons were that the owner was not available at the time of the campaign (26.5%) and that owner failed to restrain his/her dog(s) (23.8%) (S8 Table).

### Rabies knowledge in dog owners

In the household survey, 75.4% (*n* = 333; including five unavailable answers) of dog owners answered that he/she was knowledgeable about “rabies.” The main sources of their knowledge on rabies were from their family, relatives/neighbors, and through their experiences from keeping dogs (Table 6). Despite this knowledge, most of those who answered that they were knowledgeable on rabies (70.5%, *n* = 251) were unable to list the symptoms of rabies in humans. The remaining 29.5% of owners answered that they could describe the characteristic symptoms of rabies in humans. Most of the symptoms listed by the respondents as the typical symptoms of human rabies were in fact satisfactory as answers indicating actual symptoms of human rabies (Table 7). The owners who answered that they were knowledgeable about rabies were also asked about the transmission mode of rabies to humans. A total of 34.7% (*n* = 251, including one unavailable answer) of owners did not know how rabies is transmitted to humans, while 63.4% mentioned “dog bite” as a transmission mode. The remaining (approximately 1.6%) gave other answers, such as “through poison” or “by witchcraft.”

**Table 6:** Sources of information about rabies (multiple answers).

Reason	Answers (*n*)	%
Through relatives/neighbors	83	33.1
Through experience from keeping dogs/saw a rabid dog	83	33.1
Through family	80	31.9
Through TV/radio	33	13.1
Through doctors/hospitals	21	8.4
Through veterinarians/vet clinics	21	8.4
At school	12	4.8
Saw a rabid human	2	0.8
Others	3	1.2
Unavailable answers	3	1.2
Total number of answers	341	
Total number of respondents	251	

**Table 7:** Answers about symptoms of rabies in humans (multiple answers).

Reason	Answers (*n*)	%
Salivation	43	58.1
Barking like a dog	22	29.7
Getting mad (insanity)	14	18.9
Behavior change	12	16.2
Die	8	10.8
Fighting (violent)	6	8.1
Restlessness	6	8.1
Moving about	5	6.8
Mental disturbance/disorder	5	6.8
Hyperactivity	4	5.4
Biting	2	2.7
Hydrophobia	2	2.7
Crying	2	2.7
Failure eating	2	2.7
Others	5	6.8
Unavailable answers	4	5.4
Total number of answers	142	
Total number of respondents	74	

### Affordability of canine rabies vaccination and owners’ practices of dog vaccination

**Fig 2:**
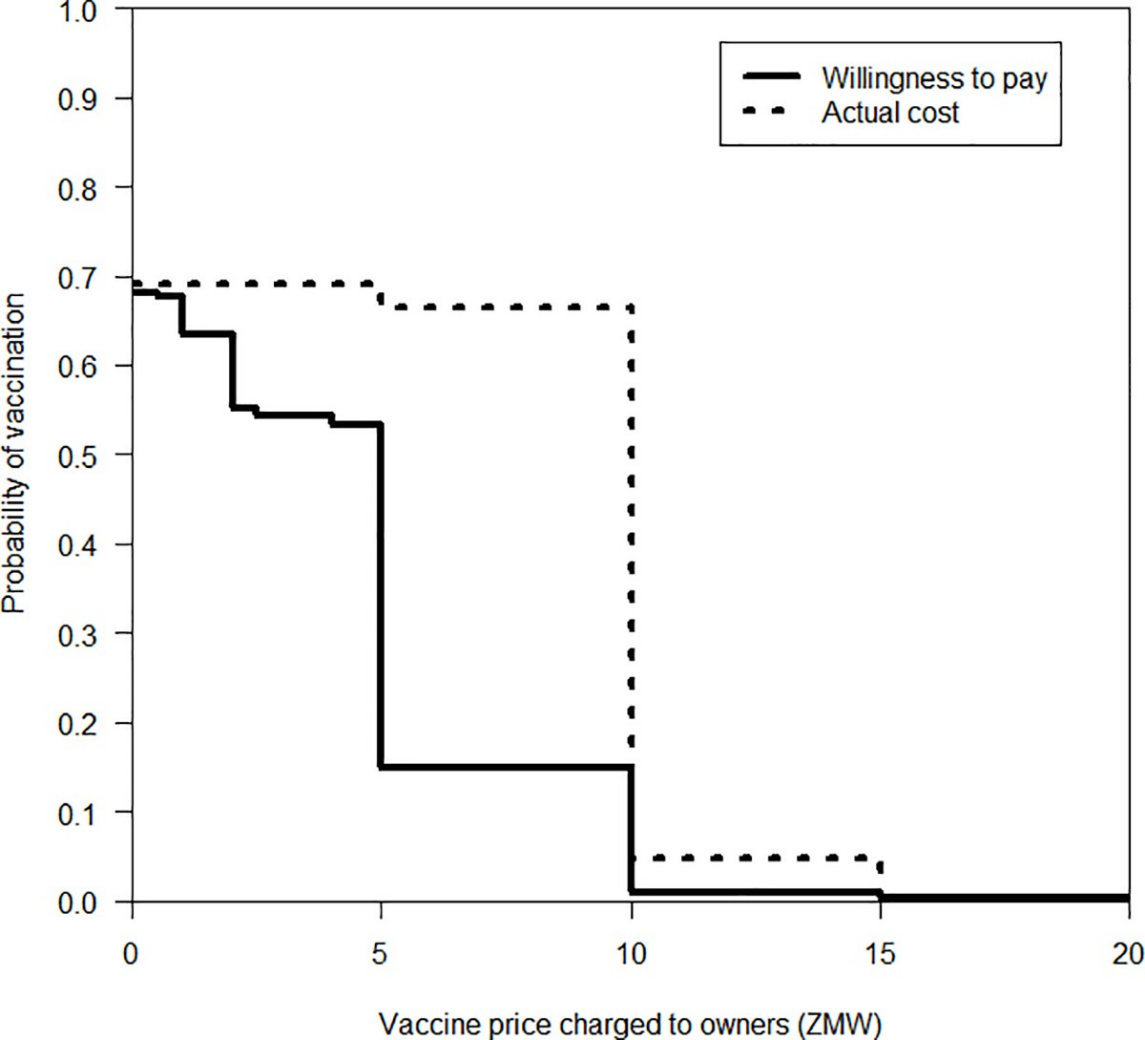
Expected probability of vaccination based on the vaccine price. The solid line shows the reverse cumulative vaccination probability curve for the amount that owners are willing to pay for a single canine rabies vaccination. The broken line shows the reverse cumulative vaccination probability curve for the amount that owners have actually paid for a single canine rabies vaccination. These vaccination probability curves are based on data collected from the household survey.

A total of 32.0% of owners desired free canine rabies vaccination, and the median amount they were willing to pay for a canine rabies vaccination was ZMW 5.00. However, 30.9% of owners had never vaccinated their dogs before, and the median amount actually paid was ZMW 10.00 (Table 8). Reverse cumulative vaccination probability curves created based on the aforementioned data highlighted significant differences in the decreases between the curve for willingness to pay and the curve for the actual amount paid (*p* < 0.05, Fig 2). Regarding the owners’ practices of vaccinating their dogs, 86.8% of dog owners who had their dogs vaccinated in the past (*n* = 234, including 16 unavailable answers) only did so when the veterinary officers came to their villages. A total of 3.4% of owners said that they vaccinated their dogs at home while another 1.7% and 0.9% vaccinated their dogs at the DVO and during mass vaccination campaigns, respectively.

**Table 8:** Affordability of canine rabies vaccination.

(ZMW)	Number of responses (%)	
	Willingness to pay	Actual cost
0 (or never vaccinated before)	105 (32.0)	99 (30.9)
0.50–5.00	174 (53.0)	8 (2.5)
10.00	46 (14.0)	198 (61.9)
15.00	2 (0.6)	14 (4.4)
20.00	1 (0.3)	1 (0.3)
Total number of valid responses	328	320
Unavailable answers	5	13

ZMW (Zambian kwacha): 1 USD was equivalent to ZMW 10.36 on May 27, 2016

## Discussion

This paper describes a canine mass rabies vaccination campaign in the rural parts of Mazabuka District in Zambia and how such a program can lead to success. This is the first report estimating vaccination coverage after a mass vaccination campaign in rural Zambia.

The present study provides information on the local dog population and its demographics in the chosen study area. In agreement with earlier studies from other rabies endemic countries in Africa and Asia [10,28,38–43], the studied dog population in the rural part of Mazabuka was young and male-biased. This male-biased sex ratio may be a result of the owners’ preference of male dogs for various roles (e.g., better guard dogs) [12] and the higher mortality rate in female dogs [9]. In accordance with the relatively low survivorship in female dogs reported frequently [38,40], this study also supported the tendency of lower survival in females than in males. Almost all dogs in the study area were kept for the purposes of security as guard dogs, followed by hunting purposes. Because our study area is located comparatively near national parks and game management areas in the Kafue flats, it is highly possible that hunting dogs frequently come into contact with wild animals.

The human-to-dog ratio was determined to be 4.45:1 in the study area. Earlier studies reported the human-to-dog ratio in Zambia to be 45:1 in the urban Lusaka District [26], 6.7:1 in a semi-rural setting in Chongwe District, Lusaka Province [26], and 3.0:1 in Nyimba District, a rural setting [27]. It is generally understood that the human-to-dog ratios in rural settings are lower than those in urban settings [6,38–41,44]. This is possibly associated with the fact that dog density in rural settings is generally lower than that in urban settings given the tendency for rural settings to allow residents to have more dogs. Focusing on the human-to-dog ratio in rural settings, the ratios recorded in rural Zambia (the present study and the study in Nyimba District [27]) are lower than the ratios recorded in other rural settings of African countries [6,38,40]. Although the factors contributing to this lower human-to-dog ratio in rural Zambia have not been clarified, this simply signifies that the dog population per human population tends to be larger in rural Zambia compared with other rural settings of African countries. This implies that opportunities to contact dogs per person might increase in rural Zambia.

The population of ownerless dogs in the study area was estimated to be very low compared with the population of owned dogs. This suggests that rabies control in humans and dogs is feasible through mass vaccination campaigns targeting owned dogs. The overall vaccination coverage achieved in the first mass vaccination campaign was estimated to range between 19.8% and 51.6% in each targeted dog population. Vaccination coverage of the owned dog population of 20.9% to 52.6% were attained in the four study zones. These figures are still lower than the 70% vaccination coverage recommended by WHO that should be achieved in mass vaccination campaigns [4,11,45]; it is also below the vaccination coverage reported earlier through free mass vaccination campaigns conducted in other African countries: in urban settings in N’Djaména, Chad [31], and Iringa, Tanzania [39], and in rural settings in the Serengeti [6] and the Mara Region [5] in Tanzania. As for Zambia, reports showing that sufficient vaccination coverage could be attained through central-point mass vaccination in rural areas are lacking. Mulipukwa et al. reported a vaccination coverage of 8.7% based on a household survey among owned dogs in Nyimba District, Eastern Province [27]. This figure was similar to, but still higher than, the pre-coverage figure before our mass vaccination, which was roughly estimated based on the data of our household survey (3.9% in zone A, 4.0% in zone B, 0% in zone C, and 7.5% in zone D). This finding implies that ordinary vaccination coverage in dog populations of Zambia, particularly in rural settings, is considerably less than the critical threshold coverage of 20–45% required to interrupt rabies transmission in a dog population [8].

The following three major reasons were given for non-participation in the first mass vaccination campaign: lack of information, owners’ unavailability, and owners being unable to handle their dogs. Despite putting up posters at major gathering points where they could be seen by the public one week before the day of the first mass vaccination, and traveling by motorcycles with loudspeakers in the target zones several times during the week prior to the vaccination, almost one-third of non-participating owners stated that they were not informed about the campaign. First, this simply indicates that such advertisements were not sufficient to reach all dog owners. This was likely because of the increased numbers of posters that are usually displayed in public places advertising all sorts of things that might not be appealing to all community members and because of the limited timing and frequency of the publicity by motorcycles using loudspeakers. Zone D had a much larger area and more spread out houses (i.e., not along main streets) than the other zones, and this could have reduced the probability for dog owners and other members of the community to read and spread the information on the rabies vaccination campaign, ultimately resulting in a notably lower coverage. Secondly, dog owners frequently reported that they had not been informed about the mass vaccination [28,46], and this may be the easiest answer to provide without admitting their actual reasons for non-participation. Thirdly, the coverage after the follow-up mass vaccination campaign also failed to reach 70%, as recommended by WHO, in zones C and D even though all households involved in the household survey had received the flyers. A possible influencing factor was the day on which the mass vaccination campaign was conducted in zones C and D (Sunday), although the actual reasons for owners’ non-participation and the relationship between choice of day and owners’ non-participation were unclear. This, however, implies the limit of enhancing vaccination coverage in the current conditions (e.g., arrangement of day and time and the owners’ low prioritization of vaccination). Furthermore, this may be related to the reason of “owners’ unavailability,” which was the second major reason for non-participation, and it indicates that owner-related scheduling conflicts limit the amount of vaccination coverage achievable. Fundamentally increasing dog owners’ awareness of the importance of canine rabies vaccination, which is also related to the owners’ knowledge on rabies mentioned in the latter paragraph, is necessary to obtain the maximum outcome of mass vaccination campaigns. Additionally, promoting community support involving other stakeholders such as local chiefs, local veterinarians and human doctors, and local teachers at schools is essential to achieve a successful mass vaccination campaign. Dog handling difficulties was the third major reason owners cited for not participating in the mass vaccination. Most of the owned dogs in the target zones were allowed to roam freely, as is common in most other African countries. Our findings were similar to earlier reports on free mass vaccinations in other African countries [31,46] and indicate that improvement in owners’ dog handling skills, general dog training knowledge, and proper equipment use (e.g., collar and chain) are still required. An alternative vaccine delivery strategy of house-to-house vaccination has been recommended in cases where dogs are difficult to handle. In this program, the owners do not have to take their dogs to long-distance vaccination sites, albeit this approach involves substantial labor and capital investments [6]. This strategy is also applicable in extremely remote communities, as discussed later. Another potential alternative strategy is oral rabies vaccination; this is a complementary measure to increase the vaccination coverage in mass parenteral dog vaccination campaigns, wherein unrestricted dogs that cannot be vaccinated parenterally under normal conditions hamper reaching 70% vaccination coverage [4]. However, regulatory authorities of different countries need to assess the suitability and necessity of the application of oral rabies vaccination for dogs considering both the benefits and the potential risks of oral vaccine-associated adverse events (particularly the limited efficacy in comparison with the parenteral vaccines, along with safety in humans and other species in cases of unintentional exposure, or release of genetically modified/self-replicating organism into the environment) [4].

There are other possible reasons that our mass vaccination campaigns did not reach the 70% vaccination coverage. First, puppies younger than three months old are not eligible for rabies vaccination in Zambia. However, puppies younger than three months old comprised 16.7% of the surveyed dog population in our study. Therefore, we propose including puppies below three months old as subjects for rabies vaccination despite the high mortality of this age class because vaccination of puppies with high-quality vaccine is strongly recommended and regarded as a cost-effective approach to maintain herd immunity [4,47,48]. Second, the comparatively large area of zone D may have reduced the owners’ motivation to take their dogs to the vaccination sites. It has previously been reported that vaccination coverage decreased as household distance from the vaccination site increased [6], but this early study noted that the coverage was generally greater than 70% even at 5 km from the vaccination sites. In such cases, house-to-house vaccination combined with central-point mass vaccination will be applicable although it requires substantial investment in labor and capital and is operationally difficult [6]. However, the continuous shortage of veterinary field staff and resources for rabies vaccination in Zambia [27] are obstacles to overcome for the application of house-to-house vaccination.

Although most of the dog owners considered themselves knowledgeable about rabies, the majority did not in fact know the symptoms of rabies in humans. Moreover, approximately one-third of them did not know the transmission mode of rabies. These data imply that dog owners may not have sufficiently accurate knowledge on rabies, even if they have heard the term before. More official education about rabies from relevant authorities (e.g., government, medical hospitals, and veterinary clinics) could be utilized to acquire correct knowledge. This would enhance the public awareness of rabies, which could lead to a better understanding of responsible dog ownership coupled with the importance of canine vaccination. The official education from experts could also provide people with proper skills to better handle their dogs. These are all steps that could help increase vaccination coverage to a point that can be effective in controlling or avoiding rabies outbreaks.

Dog owners’ willingness to pay for the rabies vaccine is another consideration when promoting canine vaccination. Our data show that the median price of rabies vaccines in rural Mazabuka District was ZMW 10.00, but owners felt burdened paying that much for the vaccination. Free vaccination will be necessary to attain vaccination coverage of 70% or higher. In our study area, canine vaccination is commonly distributed by personnel from the DVO by visiting villages. This visiting-community campaign method is thought to be appropriate for remote rural areas far from veterinary clinics or DVO headquarters [6]. However, there is evidence that the pre-vaccination coverage in our study area was roughly 0–7.5% according to the results from our household survey. This may indicate the owners’ reluctance to vaccinate, or it may have been caused by a variety of other factors, such as the owners not being provided enough chances to receive vaccination, which could have been caused by a lack of resources [27]. By ensuring opportunities for owners to have their dogs vaccinated based on regular enforcement by administering and providing free vaccinations, vaccination coverage could be improved, resulting in enhancing public health and maintaining herd immunity.

According to the Leslie matrix, the dog population growth rate was estimated at 15% per annum (*λ* = 1.15). The instantaneous rate of increase, *r*, was calculated as 0.14. These values, which indicate high population growth, are similar to other reports demonstrating the growth of dog populations in African countries [38–41]. The main determinants of population growth were the survival of younger age classes. Although this dog population had a high mortality of almost 50% in dogs under one year old, this mortality was lower compared with those in earlier reports conducted in Iringa of Tanzania (72%) [39] and Bamako of Mali (73%) [41]. Assuming a vaccination coverage of 70% attained at the start of the year, the data obtained from our survey indicated that the coverage would decrease to 43.7% in one year because of the death of vaccinated dogs and the birth of naïve juveniles under this level of population growth. Based on the critical vaccination threshold of 20–45% that should be maintained to prevent rabies outbreaks [24], annual vaccination campaigns might be sufficient in this dog population if 70% of the population is vaccinated at the start of a year. As mentioned above, information on dog demographics provides beneficial parameters for designing and planning canine rabies mass vaccinations. The present study highlighted some parameters for evaluating population demographics and growth projections of dog populations in rural Zambia; these parameters can be utilized for designing and planning canine mass vaccinations. However, the static life table and the Leslie matrix used in the present study are limited because they do not take migration or density effects into account. Recently, longitudinal cohort studies have revealed that no population growth was observed in domestic dog populations in rabies endemic countries [9,12]. Conversely, a decline in population was observed in some areas in previous studies [9,12]. These earlier studies demonstrated that the high birth and death rates resulting in high turnover of the population rather than net population growth lead to the decline of vaccination coverage in the dog populations in rabies endemic countries [9,12]. The present study did not perform longitudinal monitoring of the population dynamics that can be used to investigate birth and death rates and dog migrations. This is a limitation of our study because of its cross-sectional nature. To obtain more realistic evaluations and projections, cohort studies that take dog migration (movement of dogs by humans) that consists of a substantial fraction of a dog population into account must be conducted [9,12,41]. From the viewpoints of designing and implementing effective canine mass vaccinations in Zambia, as we revealed in our survey, we propose performing annual canine rabies mass vaccinations and including puppies below three months old in the vaccination campaign to attain the 70% threshold coverage in a dog population.

## Conclusions

This study is the first report on rural dog demographics and canine vaccination coverage attained by conducting a free mass vaccination campaign in Zambia; it also provides an estimate of the ownerless dog population in the rural part of Zambia. This study indicated that the number of ownerless dogs was quite low compared with the number of owned dogs in a rural setting in Zambia. Thus, there is a potential to control rabies through canine mass vaccination campaigns targeted at owned dogs, although the first mass vaccination campaign attained only low vaccination coverage. To achieve the 70% coverage recommended by WHO, we propose including puppies younger than three months old in rabies vaccination programs. Although puppies are currently not included in rabies vaccination in Zambia, the puppy population is not negligible and would be necessary to attain the 70% coverage and obtain the maximum outcome of rabies mass vaccination. This study also suggests that increasing education on rabies and its control, responsible dog ownership, good dog handling, and mass vaccination campaigns are necessary for dog owners to achieve a higher vaccination coverage. Moreover, better advertising to and education of the community (particularly the key community leaders such as local chiefs, teachers, and others) on the importance of rabies and responsible dog ownership cannot be overemphasized to ensure the promotion and sustainability of the rabies mass vaccination campaigns. Furthermore, our study re-emphasized that regular annual canine rabies mass vaccinations are necessary to secure owners with vaccination opportunities and to maintain protective herd immunity among dogs. In conclusion, this study highlighted the potential for controlling rabies in rural parts of Zambia and identified key issues that require attention for the success of future rabies campaign/control programs.

## Supporting information

S1 Appendix Questionnaire sheet. (PDF)

S2 Appendix Prior distribution for parameters. (DOCX)

S3 Appendix Scripts for the model in OpenBUGS. (DOCX)

S4 Appendix Formulas for calculating the life history parameters. (DOCX)

S1 Table Prior distributions. (DOCX)

S2 Table Overall population demographics (Static life table). (DOCX)

S3 Table Female fecundity. (DOCX)

S4 Table Sex-specific population demographics (Static life tables). (DOCX)

S5 Table Posterior distributions. (DOCX)

S6 Table Summary of the sensitivity analysis. (XLSX)

S7 Table Proportion of vaccinated owned dogs based on the observation in the household survey. (DOCX)

S8 Table Reasons for non-participation in the first mass vaccination campaign. (DOCX)

## References

[1] Hampson K, Coudeville L, Lembo T, Sambo M, Kieffer A, Attlan M, et al. Estimating the global burden of endemic canine rabies. PLoS Negl Trop Dis. 2015;9(4):e0003709. 10.1371/journal.pntd.0003709 25881058 PMC4400070

[2] Lembo T, Hampson K, Kaare MT, Ernest E, Knobel D, Kazwala RR, et al. The feasibility of canine rabies elimination in Africa: dispelling doubts with data. PLoS Negl Trop Dis. 2010;4(2):e626. 10.1371/journal.pntd.0000626 20186330 PMC2826407

[3] Cleaveland S, Hampson K, Kaare M. Living with rabies in Africa. Vet Rec. 2007;161(9):293–294. 10.1136/vr.161.9.293 . 17766806

[4] World Health Organization. WHO Expert Consultation on Rabies. Third report. World Health Organ Tech Rep Ser. 2018;(1012):1–183. Available from: https://apps.who.int/iris/handle/10665/272364

[5] Cleaveland S, Kaare M, Tiringa P, Mlengeya T, Barrat J. A dog rabies vaccination campaign in rural Africa: impact on the incidence of dog rabies and human dog-bite injuries. Vaccine. 2003;21(17–18):1965–1973. 10.1016/s0264-410x(02)00778-8 . 12706685

[6] Kaare M, Lembo T, Hampson K, Ernest E, Estes A, Mentzel C, et al. Rabies control in rural Africa: evaluating strategies for effective domestic dog vaccination. Vaccine. 2009;27(1):152–160. 10.1016/j.vaccine.2008.09.054 18848595 PMC3272409

[7] Knobel DL, Cleaveland S, Coleman PG, Fèvre EM, Meltzer MI, Miranda ME, et al. Re-evaluating the burden of rabies in Africa and Asia. Bull World Health Organ. 2005;83(5):360–368. 15976877 PMC2626230

[8] Hampson K, Dushoff J, Cleaveland S, Haydon DT, Kaare M, Packer C, et al. Transmission dynamics and prospects for the elimination of canine rabies. PLoS Biol. 2009;7(3):e53. 10.1371/journal.pbio.1000053 19278295 PMC2653555

[9] Conan A, Akerele O, Simpson G, Reininghaus B, van Rooyen J, Knobel D. Population Dynamics of Owned, Free-Roaming Dogs: Implications for Rabies Control. PLoS Negl Trop Dis. 2015;9(11):e0004177. Epub 2015/11/06. 10.1371/journal.pntd.0004177 26545242 PMC4636342

[10] Morters MK, McKinley TJ, Horton DL, Cleaveland S, Schoeman JP, Restif O, et al. Achieving population-level immunity to rabies in free-roaming dogs in Africa and Asia. PLoS Negl Trop Dis. 2014;8(11):e3160. Epub 2014/11/13. 10.1371/journal.pntd.0003160 25393023 PMC4230884

[11] Coleman PG, Dye C. Immunization coverage required to prevent outbreaks of dog rabies. Vaccine. 1996;14(3):185–186. 10.1016/0264-410x(95)00197-9 . 8920697

[12] Morters MK, McKinley TJ, Restif O, Conlan AJ, Cleaveland S, Hampson K, et al. The demography of free-roaming dog populations and applications to disease and population control. J Appl Ecol. 2014;51(4):1096–1106. 10.1111/1365-2664.12279 25657481 PMC4285860

[13] Lee JH, Lee JB, Kim JS, Bae CS, Lee WC, Lee MJ. Review of canine rabies prevalence under two different vaccination programmes in Korea. Vet Rec. 2001;148(16):511. 10.1136/vr.148.16.511 11345995

[14] Eng TR, Fishbein DB, Talamante HE, Hall DB, Chavez GF, Dobbins JG, et al. Urban epizootic of rabies in Mexico: epidemiology and impact of animal bite injuries. Bull World Health Organ. 1993;71(5):615–624. . 8261565 PMC2393488

[15] Munang’andu HM, Mweene AS, Siamudaala V, Muma JB, Matandiko W. Rabies status in Zambia for the period 1985–2004. Zoonoses Public Health. 2011;58(1):21–27. 10.1111/j.1863-2378.2010.01368.x . 20887398

[16] Southern and Eastern African Rabies Group. Zambia country report: 2010–2012. 2013. [cited 2015 February 1]. Available from: http://searg.info/doku.php?id=aboutrabies:rabiesepidemiology:2013reportzambia

[17] Babaniyi O, Songolo P, Matapo B, Masaninga F, Mulenga F, Michelo C, et al. Epidemiological characteristics of rabies in Zambia: A retrospective study (2004–2013). Clin Epidemiol Glob Health. 2016;4(2):83–88. 10.1016/j.cegh.2016.01.003.

[18] Beyene TJ, Mourits MCM, Hogeveen H. Dog rabies data reported to multinational organizations from Southern and Eastern African countries. BMC Res Notes. 2017;10(1):199. Epub 2017/06/08. 10.1186/s13104-017-2527-7 28595654 PMC5465567

[19] Nel LH. Discrepancies in data reporting for rabies, Africa. Emerg Infect Dis. 2013;19(4):529–533. 10.3201/eid1904.120185 . 23628197 PMC3647406

[20] Cleaveland S, Fèvre EM, Kaare M, Coleman PG. Estimating human rabies mortality in the United Republic of Tanzania from dog bite injuries. Bull World Health Organ. 2002;80(4):304–310. 12075367 PMC2567765

[21] Banyard AC, Horton DL, Freuling C, Müller T, Fooks AR. Control and prevention of canine rabies: the need for building laboratory-based surveillance capacity. Antiviral Res. 2013;98(3):357–364. 10.1016/j.antiviral.2013.04.004 . 23603498

[22] Muleya W, Namangala B, Mweene A, Zulu L, Fandamu P, Banda D, et al. Molecular epidemiology and a loop-mediated isothermal amplification method for diagnosis of infection with rabies virus in Zambia. Virus Res. 2012;163(1):160–168. 10.1016/j.virusres.2011.09.010 . 21930165

[23] Röttcher D, Sawchuk AM. Wildlife rabies in Zambia. J Wildl Dis. 1978;14(4):513–517. 10.7589/0090-3558-14.4.513 . 105155

[24] Berentsen AR, Dunbar MR, Becker MS, M’soka J, Droge E, Sakuya NM, et al. Rabies, canine distemper, and canine parvovirus exposure in large carnivore communities from two Zambian ecosystems. Vector Borne Zoonotic Dis. 2013;13(9):643–649. 10.1089/vbz.2012.1233 . 23805791

[25] Muleya W, Chambaro HM, Sasaki M, Gwenhure LF, Mwenechanya R, Kajihara M, et al. Genetic diversity of rabies virus in different host species and geographic regions of Zambia and Zimbabwe. Virus Genes. 2019;55(5):713–719. Epub 2019/07/03. 10.1007/s11262-019-01682-y . 31267444

[26] De Balogh KK, Wandeler AI, Meslin FX. A dog ecology study in an urban and a semi-rural area of Zambia. Onderstepoort J Vet Res. 1993;60(4):437–443. . 7777333

[27] Mulipukwa CP, Mudenda B, Mbewe AR. Insights and efforts to control rabies in Zambia: Evaluation of determinants and barriers to dog vaccination in Nyimba district. PLoS Negl Trop Dis. 2017;11(10):e0005946. Epub 2017/10/09. 10.1371/journal.pntd.0005946 28991898 PMC5648261

[28] Durr S, Mindekem R, Kaninga Y, Doumagoum Moto D, Meltzer MI, Vounatsou P, et al. Effectiveness of dog rabies vaccination programmes: comparison of owner-charged and free vaccination campaigns. Epidemiol Infect. 2009;137(11):1558–1567. 10.1017/S0950268809002386 . 19327197

[29] Fitzpatrick MC, Hampson K, Cleaveland S, Mzimbiri I, Lankester F, Lembo T, et al. Cost-effectiveness of canine vaccination to prevent human rabies in rural Tanzania. Ann Intern Med. 2014;160(2):91–100. 10.7326/M13-0542 24592494 PMC4084874

[30] Banda R, Sandøy IF, Fylkesnes K, Janssen F. Impact of Pregnancy-Related Deaths on Female Life Expectancy in Zambia: Application of Life Table Techniques to Census Data. PLoS One. 2015;10(10):e0141689. 10.1371/journal.pone.0141689 26513160 PMC4626102

[31] Kayali U, Mindekem R, Yémadji N, Vounatsou P, Kaninga Y, Ndoutamia AG, et al. Coverage of pilot parenteral vaccination campaign against canine rabies in N’Djaména, Chad. Bull World Health Organ. 2003;81(10):739–744. 14758434 PMC2572337

[32] Chapman DG. Some properties of the hypergeometric distribution with applications to zoological sample censuses. Berkeley: University of California Press; 1951.

[33] Seber GAF. The Effects of Trap Response on Tag Recapture Estimates. Biometrics. 1970;26(1):13–22. 10.2307/2529040

[34] Tenzin T, McKenzie JS, Vanderstichel R, Rai BD, Rinzin K, Tshering Y, et al. Comparison of mark-resight methods to estimate abundance and rabies vaccination coverage of free-roaming dogs in two urban areas of south Bhutan. Prev Vet Med. 2015;118(4):436–448. 10.1016/j.prevetmed.2015.01.008 25650307

[35] Caughley G. Mortality. In: Analysis of Vertebrate Populations. New Jersey: Blackburn Press; 1977. pp. 85–106.

[36] Pianka ER. Vital statistics of population. In: Evolutionary ecology. 6th edition. San Francisco: Benjamin Cummings; 1999. pp. 134–176.

[37] R Core Team. R: A language and environment for statistical computing. Vienna, Austria.: R Foundation for Statistical Computing; 2020.

[38] Kitala P, McDermott J, Kyule M, Gathuma J, Perry B, Wandeler A. Dog ecology and demography information to support the planning of rabies control in Machakos District, Kenya. Acta Trop. 2001;78(3):217–230. 10.1016/s0001-706x(01)00082-1 . 11311185

[39] Gsell AS, Knobel DL, Kazwala RR, Vounatsou P, Zinsstag J. Domestic dog demographic structure and dynamics relevant to rabies control planning in urban areas in Africa: the case of Iringa, Tanzania. BMC Vet Res. 2012;8:236. 10.1186/1746-6148-8-236 23217194 PMC3534358

[40] Czupryna AM, Brown JS, Bigambo MA, Whelan CJ, Mehta SD, Santymire RM, et al. Ecology and Demography of Free-Roaming Domestic Dogs in Rural Villages near Serengeti National Park in Tanzania. PLoS One. 2016;11(11):e0167092. Epub 2016/11/28. 10.1371/journal.pone.0167092 27893866 PMC5125679

[41] Mauti S, Traoré A, Sery A, Bryssinckx W, Hattendorf J, Zinsstag J. First study on domestic dog ecology, demographic structure and dynamics in Bamako, Mali. Prev Vet Med. 2017;146:44–51. Epub 2017/07/21. 10.1016/j.prevetmed.2017.07.009 . 28992927

[42] Estrada R, Vos A, De Leon R, Mueller T. Field trial with oral vaccination of dogs against rabies in the Philippines. BMC Infect Dis. 2001;1:23. Epub 2001/11/28. 10.1186/1471-2334-1-23 11737869 PMC60992

[43] Matter HC, Wandeler AI, Neuenschwander BE, Harischandra LP, Meslin FX. Study of the dog population and the rabies control activities in the Mirigama area of Sri Lanka. Acta Trop. 2000;75(1):95–108. 10.1016/s0001-706x(99)00085-6 . 10708011

[44] Gibson AD, Handel IG, Shervell K, Roux T, Mayer D, Muyila S, et al. The Vaccination of 35,000 Dogs in 20 Working Days Using Combined Static Point and Door-to-Door Methods in Blantyre, Malawi. PLoS Negl Trop Dis. 2016;10(7):e0004824. Epub 2016/07/14. 10.1371/journal.pntd.0004824 27414810 PMC4945057

[45] World Health Organization. Guidelines for dog rabies control. World Health Organization. 1987; Contract No.: VPH/83.43 Rev. 1. Available from: https://www.who.int/rabies/en/Guidelines_for_dog_rabies_control.pdf

[46] Muthiani Y, Traoré A, Mauti S, Zinsstag J, Hattendorf J. Low coverage of central point vaccination against dog rabies in Bamako, Mali. Prev Vet Med. 2015;120(2):203–209. 10.1016/j.prevetmed.2015.04.007 . 25953653

[47] Morters MK, McNabb S, Horton DL, Fooks AR, Schoeman JP, Whay HR, et al. Effective vaccination against rabies in puppies in rabies endemic regions. Vet Rec. 2015;177(6):150. Epub 2015/06/24. 10.1136/vr.102975 26109286 PMC4552936

[48] Anderson A, Kotzé J, Shwiff SA, Hatch B, Slootmaker C, Conan A, et al. A bioeconomic model for the optimization of local canine rabies control. PLoS Negl Trop Dis. 2019;13(5):e0007377. 10.1371/journal.pntd.0007377 31116732 PMC6548399

