# Supplementary material for: Domestic dog demographics and estimates of canine vaccination coverage in a rural area of Zambia for the elimination of rabies
Source: PLoS Negl Trop Dis. 2021 Apr 28;15(4):e0009222. doi: 10.1371/journal.pntd.0009222 (PMC8081203; doi:10.1371/journal.pntd.0009222)
Supplement: S1 Appendix — (PDF) [file pntd.0009222.s001.pdf]

SURVEY OF DOG DEMOGRAPHICS AND VACCINATION STATUS

This is the survey regarding the situation of dog demographics and vaccination.
The information obtained from this survey will be utilized for scientific research only.

School of Veterinary Medicine, The University of Zambia, LUSAKA
Hokkaido University Center for Zoonosis Control in Zambia

A. PARTICULAR OF DOG OWNER (INTERVIEWEE) [**BLOCK LETTER]**

1. **Name of owner (Interviewee)** ....
2. **Residential address** ....
3. **Contact Number** ....
4. **Sex** ☐ Male / ☐ Female   5. **Age** ....   6. **Occupation** ....
7. **How many persons do you have in your household?** .... **persons**

   Number of Adults: ....   **Number of Children (under 18 years old):** ....
8. **How many dogs do you have in your household (including puppies)?** .... **dogs**
9. **Please specify the details of all of your dogs such as sex, age and vaccination status below.**

<u>If the dog is unmarked or unidentified</u>, check the previous vaccine certificate

Dog	Age	Sex	Spray-mark on the body	Vaccination status	Date of the previous (latest) vaccination	Batch No. of the vaccine	Valid for
1	☐☐ ☐☐<br>Y Y year M M months	☐Male<br>☐Female<br>☐*Sterilized*	☐Marked<br>☐Unmarked<br>☐Unidentified	☐Yes<br>☐No	☐☐☐☐☐☐☐☐<br>D D M M Y Y Y Y		☐☐ / ☐☐<br>M M months / Y Y year
2	☐☐ ☐☐<br>Y Y year M M months	☐Male<br>☐Female<br>☐*Sterilized*	☐Marked<br>☐Unmarked<br>☐Unidentified	☐Yes<br>☐No	☐☐☐☐☐☐☐☐<br>D D M M Y Y Y Y		☐☐ / ☐☐<br>M M months / Y Y year
3	☐☐ ☐☐<br>Y Y year M M months	☐Male<br>☐Female<br>☐*Sterilized*	☐Marked<br>☐Unmarked<br>☐Unidentified	☐Yes<br>☐No	☐☐☐☐☐☐☐☐<br>D D M M Y Y Y Y		☐☐ / ☐☐<br>M M months / Y Y year
4	☐☐ ☐☐<br>Y Y year M M months	☐Male<br>☐Female<br>☐*Sterilized*	☐Marked<br>☐Unmarked<br>☐Unidentified	☐Yes<br>☐No	☐☐☐☐☐☐☐☐<br>D D M M Y Y Y Y		☐☐ / ☐☐<br>M M months / Y Y year
5	☐☐ ☐☐<br>Y Y year M M months	☐Male<br>☐Female<br>☐*Sterilized*	☐Marked<br>☐Unmarked<br>☐Unidentified	☐Yes<br>☐No	☐☐☐☐☐☐☐☐<br>D D M M Y Y Y Y		☐☐ / ☐☐<br>M M months / Y Y year
6	☐☐ ☐☐<br>Y Y year M M months	☐Male<br>☐Female<br>☐*Sterilized*	☐Marked<br>☐Unmarked<br>☐Unidentified	☐Yes<br>☐No	☐☐☐☐☐☐☐☐<br>D D M M Y Y Y Y		☐☐ / ☐☐<br>M M months / Y Y year
7	☐☐ ☐☐<br>Y Y year M M months	☐Male<br>☐Female<br>☐*Sterilized*	☐Marked<br>☐Unmarked<br>☐Unidentified	☐Yes<br>☐No	☐☐☐☐☐☐☐☐<br>D D M M Y Y Y Y		☐☐ / ☐☐<br>M M months / Y Y year
8	☐☐ ☐☐<br>Y Y year M M months	☐Male<br>☐Female<br>☐*Sterilized*	☐Marked<br>☐Unmarked<br>☐Unidentified	☐Yes<br>☐No	☐☐☐☐☐☐☐☐<br>D D M M Y Y Y Y		☐☐ / ☐☐<br>M M months / Y Y year
9	☐☐ ☐☐<br>Y Y year M M months	☐Male<br>☐Female<br>☐*Sterilized*	☐Marked<br>☐Unmarked<br>☐Unidentified	☐Yes<br>☐No	☐☐☐☐☐☐☐☐<br>D D M M Y Y Y Y		☐☐ / ☐☐<br>M M months / Y Y year

If you have more dogs, please write down the details in the spare form.

FOR STAFF USE ONLY

Date of sampling	***Area***	***Specimen No.***
☐☐☐☐☐☐☐☐<br>D D M M Y Y Y Y		☐☐☐☐

B. FECUNDITY OF FEMALE DOGS

If you have female dog(s), describe the details of reproductive history.

Fem-ale Dog	Current age	Total number of litters (delivery) in lifetime	Number of litters (delivery) in the last 12 months	Size of the latest litter	Fate of puppies in the latest litter
1	☐☐☐☐<br>Y Y M M			Total<br>☐ of male puppies<br>☐ of female puppies	☐ of them were kept in own house.<br>(☐ males and ☐ females)<br><br>☐ of them were sold/given away within the village.<br>(☐ males and ☐ females at ☐ months old)<br>(☐ males and ☐ females at ☐ months old)<br><br>☐ of them were sold/given away out of the village.<br>(☐ males and ☐ females at ☐ months old)<br>(☐ males and ☐ females at ☐ months old)<br><br>☐ of them died.<br>(☐ males and ☐ females at ☐ months old)<br>(☐ males and ☐ females at ☐ months old)
2	☐☐☐☐<br>Y Y M M			Total<br>☐ of male puppies<br>☐ of female puppies	☐ of them were kept in own house.<br>(☐ males and ☐ females)<br><br>☐ of them were sold/given away within the village.<br>(☐ males and ☐ females at ☐ months old)<br>(☐ males and ☐ females at ☐ months old)<br><br>☐ of them were sold/given away out of the village.<br>(☐ males and ☐ females at ☐ months old)<br>(☐ males and ☐ females at ☐ months old)<br><br>☐ of them died.<br>(☐ males and ☐ females at ☐ months old)<br>(☐ males and ☐ females at ☐ months old)
3	☐☐☐☐<br>Y Y M M			Total<br>☐ of male puppies<br>☐ of female puppies	☐ of them were kept in own house.<br>(☐ males and ☐ females)<br><br>☐ of them were sold/given away within the village.<br>(☐ males and ☐ females at ☐ months old)<br>(☐ males and ☐ females at ☐ months old)<br><br>☐ of them were sold/given away out of the village.<br>(☐ males and ☐ females at ☐ months old)<br>(☐ males and ☐ females at ☐ months old)<br><br>☐ of them died.<br>(☐ males and ☐ females at ☐ months old)<br>(☐ males and ☐ females at ☐ months old)

If the space is not enough, use the spare form to describe delivery histories for all of your female dogs.

Continue on the next page

C. QUESTIONS

Please tick (✓) where applicable.

1. What is your dog(s) for?
   ☐Pet ☐Guard dog
   ☐Hunting: Please specify the type of game hunted: .... ☐Other ....

2. How do you keep your dog usually?
   ☐Complete free-range ☐Free-range only within the fenced owner's premise ☐Confine inside cage
   ☐Confine with chain ☐Other ....

3. How much time do your dogs spend outside of your premises in a day?
   ☐All day long ☐Not at all ☐Only at night (From : to :)
   ☐Only in daytime (From : to :) ☐Other: Please specify the duration ...... hours

4. **If you did not bring your dog(s) to the free mass vaccination held on 21st – 22nd May,** kindly explain why you did not participate in the mass vaccination:

   ....

5. How much are you willing to pay for dog vaccination per capita?
   .... Kwacha

6. How much do you usually pay for dog vaccination per capita?
   .... Kwacha

7. Where do you go to vaccinate your dog(s) usually?
   ☐Mazabuka DVO Headquarter ☐The nearest vet camp
   ☐Private vet clinic: Specify the name of the clinic .... ☐Others ....

8. Do you know the disease of "rabies"?
   ☐Yes ☐No

9. **If your answer is "yes" in the question 8,** how did you know about rabies?
   ☐Through family ☐Through relatives/neighbour ☐At school ☐Through a doctor/at hospital
   ☐Through a veterinarian/at veterinary office ☐Through TV/Radio ☐Other ....

10. **If your answer is "yes" in the question 8,** do you know the characteristic symptoms of human rabies cases?
    ☐Yes, I do. The symptom(s): ....
    ☐No, I do not know.

11. **If your answer is "yes" in the question 8,** do you know the transmission route of rabies to human?
    ☐Yes, I do. The transmission route: ....
    ☐No, I do not know.

Continued on the next page

C. QUESTIONS (Continuation)

Please tick (✓) where applicable.

12. Was there any person bitten by dog or other animal **in your household in the last 12 months**?
    - ☐Yes ▪▪▪Please specify the relation of the bitten person with you ....
    - ☐No

13. What was he/she bitten by at the time?
    - ☐Dog kept in own household ☐Dog kept in the other household ☐Feral (Stray) dog
    - ☐Other animal: Specify ....

14. Which part was he/she bitten on at the time?
    - ☐Head ☐Body trunk ☐Arms ☐Legs

15. Did he/she receive **anti-rabies vaccination** after the bite?
    - ☐Yes ☐No

<u>For the interviewer</u>

1. Is the household completely surrounded by a brick wall/wire fence or others? **☐Yes / ☐No**
2. Are the dog(s) in the household completely confined (by chain / inside cage etc.) or not? **☐Yes / ☐No**

Specimen No.

