# Supplementary material for: Domestic dog demographics and estimates of canine vaccination coverage in a rural area of Zambia for the elimination of rabies
Source: PLoS Negl Trop Dis. 2021 Apr 28;15(4):e0009222. doi: 10.1371/journal.pntd.0009222 (PMC8081203; doi:10.1371/journal.pntd.0009222)
Supplement: S2 Appendix — (DOCX) [file pntd.0009222.s002.docx]

**S2 Appendix. Prior distribution for parameters**

This appendix mainly refers to the methodology described by Kayali et al.[1].

**The parameters *N_i_* and *a_i_* for estimating ownerless dog population.** The parameter *N_i_* is the total number of ownerless dogs in zone *i*. The parameter *N_i_* was expressed as a fraction of the total owned dogs (in particular the mean and variance), that is *N_i_* = *a_i_M_i_*. Uniform prior distributions were assumed for *a_i_*, *a_i_* ~ *U* (0, 0.3) in each zone. The parameter of the above uniform distribution was chosen by roughly estimating the ownerless dog population in the study zone based on the data obtained from the household questionnaire.

**Recapture probability *p_i_*.** To estimate the recapture probability *p_i_*, we decomposed *p_i_* to three parameters. The parameter *p_i_* was obtained by the product of coverage (*C_i_*), encountering (*E_i_*) and recording (*R_i_*): the area covered by the transect line (coverage), the probability to encounter a specific dog provided the area is covered by the transect (encountering), and the probability of the observer to actually record an encountered dog (recording). For each component, uniform priors were adopted as explained below and shown in S1 Table. The lower limit for the coverage was obtained by dividing the area covered by the transect (allowing 25 m along each side of the line to include a part of the road as well as the yard of the compound next to the road) by the total area of the zone. The upper limit for the coverage was calculated by dividing the total length of the actual transect lines by the total length of roads (including small pathways) in the study zone. This was because most households were along the streets (or has small pathways from the main street to their houses) in the study zones, moreover, each target zone had vast extent of cotton and/or maize fields in which dogs were rarely around to be seen. The limits of the uniform prior for the encountering component are based on our observation that many dogs gather around their premises and could therefore be seen. We concluded that recording was very high by comparing the counts of dogs recorded by the two observers who traveled together along each transect line.

**Confinement probability *c*_1,_*_i_* and *c*_2,_*_i_*.** As for the prior distribution of the confinement probabilities *c*_1,_*_i_* and *c*_2,_*_i_*, beta distributions were employed. Based on the household survey, we set the proportion itself, and the standard error of the proportion of dogs that spend no time outside the premises and were on the premises surrounded/closed completely by wire fences/brick walls as the mean and the standard deviation of prior distribution of *c*_1,_*_i_* and *c*_2,_*_i_*. S1 Table shows the prior distributions of confinement probabilities.

The posterior distributions for the parameters explained above are shown in S5 Table.

**Reference**

1. Kayali U, Mindekem R, Yémadji N, Vounatsou P, Kaninga Y, Ndoutamia AG, et al. Coverage of pilot parenteral vaccination campaign against canine rabies in N'Djaména, Chad. Bull World Health Organ. 2003;81(10):739-744. PubMed PMID: 14758434; PubMed Central PMCID: PMC2572337.
