# Supplementary material for: Domestic dog demographics and estimates of canine vaccination coverage in a rural area of Zambia for the elimination of rabies
Source: PLoS Negl Trop Dis. 2021 Apr 28;15(4):e0009222. doi: 10.1371/journal.pntd.0009222 (PMC8081203; doi:10.1371/journal.pntd.0009222)
Supplement: S3 Appendix — (DOCX) [file pntd.0009222.s003.docx]

**S3 Appendix. Scripts for the model in OpenBUGS**

#We basically followed the model below:

#3 chains, Burn in 20000, 40000 iterations, without thinning

model {

for (i in 1:zone) {

for( t in 1 : T ) {

# number of recaptured marked (owned) dogs during the transect survey

x1[i,t]~dbin(p[i,t],n1[i])

# number of recaptured unmarked (owned + ownerless) dogs during the transect survey

z[i,t]~dbin(p[i,t],n2[i])

# number of recaptured unmarked (owned) dogs during the transect survey

x2[i,t]~dbin(p[i,t],n3[i])

p[i,t]~dunif(pmin[i],pmax[i]) #recapture probability

}

n1[i]<-round((1-c1[i])*Mv[i])

n2[i]<-round((1-c2[i])*(M[i]-Mv[i])+N[i])

n3[i]<-round((1-c2[i])*(M[i]-Mv[i]))

M[i]~dnorm(mean_m[i], Mtau[i])

Mtau[i]<-1/Mvar[i]

a[i]~dunif(0, 0.3) #ratio of ownerless dogs to owned dogs

N[i]<-a[i]*M[i] # number of ownerless dogs

c1[i]~dbeta(a1[i],b1[i]) #confinement probability of marked owned dogs

c2[i]~dbeta(a2[i],b2[i]) #confinement probability of unmarked owned dogs

#vaccination coverage in the owned dog population through the 1st mass vaccination campaign

vc1[i]<-Mv[i]/M[i]

#vaccination coverage in the total dog population through the 1st mass vaccination campaign

vc2[i]<-Mv[i]/(M[i]+N[i])

#vaccination coverage in the owned dog population through the 1st and follow-up mass vaccination campaigns

vc1sec[i]<-(Mv[i]+Mv2[i])/M[i]

#vaccination coverage in the total dog population through the 1st and follow-up mass vaccination campaigns

vc2sec[i]<-(Mv[i]+Mv2[i])/(M[i]+N[i])

# Parameters of the Beta distribution assigned to the confinement probabilities of marked (c1) and unmarked (c2) owned dogs. They expressed in terms of the mean and variances

a1[i]<-m1[i]*m1[i]*((1-m1[i])/(s1[i]*s1[i]))-m1[i]

b1[i]<-a1[i]*(1-m1[i])/m1[i]

a2[i]<-m2[i]*m2[i]*((1-m2[i])/(s2[i]*s2[i]))-m2[i]

b2[i]<-a2[i]*(1-m2[i])/m2[i]

}

}

list(

T=1, #Number of transects per zone

# number of recaptured marked (owned) dogs during the transect survey

x1=structure(.Data=c(yy, yy, yy, yy),.Dim=c(4,1)),

# number of recaptured unmarked (owned + ownerless) dogs during the transect survey

z=structure(.Data=c(yy, yy, yy, yy),.Dim=c(4,1)),

zone=4, #Number of study zones

m1=c(yy, yy, yy, yy),

s1=c(yy, yy, yy, yy),

m2=c(yy, yy, yy, yy),

s2=c(yy, yy, yy, yy),

Mv=c(yy, yy, yy, yy),

mean_m=c(yy, yy, yy, yy),

Mvar=c(yy, yy, yy, yy),

pmin=c(yy, yy, yy, yy),

pmax=c(yy, yy, yy, yy),

Mv2=c(yy, yy, yy, yy)

)

#Mv: number of vaccinated owned dogs during the 1st mass vaccination campaign

#Mv2: number of vaccinated owned dogs during the follow-up mass vaccination campaign

#mean_m: total number of owned dogs estimated by the Chapman estimate formula

#Mvar: variance of the total number of owned dogs estimated by the Chapman estimate formula

#m1 and s1: mean and standard deviation of the prior distribution of the confinement probability (c1)

#m2 and s2: mean and standard deviation of the prior distribution of the confinement probability (c2)

#pmin and pmax: prior parameters of a uniform distribution for the recapture probability

#yy should be replaced by the actual data

#No confinement of marked and unmarked owned dogs (pertaining to c1 and c2, respectively) was observed in zone B, and no confinement of unmarked owned dogs (pertaining to c2) was observed in zones C and D. Therefore, the model in the following pages was actually used one by one for each study zone because c1 or c2 was not defined in the model above for the study zone where no confinement was observed for marked or unmarked owned dogs.

#Zone A

model {

for (i in 1:zone) {

for( t in 1 : T ) {

x1[i,t]~dbin(p[i,t],n1[i])

z[i,t]~dbin(p[i,t],n2[i])

x2[i,t]~dbin(p[i,t],n3[i])

p[i,t]~dunif(pmin[i],pmax[i])

}

n1[i]<-round((1-c1[i])*Mv[i])

n2[i]<-round((1-c2[i])*(M[i]-Mv[i])+N[i])

n3[i]<-round((1-c2[i])*(M[i]-Mv[i]))

M[i]~dnorm(mean_m[i], Mtau[i])

Mtau[i]<-1/Mvar[i]

a[i]~dunif(0, 0.3)

N[i]<-a[i]*M[i]

c1[i]~dbeta(a1[i],b1[i])

c2[i]~dbeta(a2[i],b2[i])

vc1[i]<-Mv[i]/M[i]

vc2[i]<-Mv[i]/(M[i]+N[i])

vc1sec[i]<-(Mv[i]+Mv2[i])/M[i]

vc2sec[i]<-(Mv[i]+Mv2[i])/(M[i]+N[i])

a1[i]<-m1[i]*m1[i]*((1-m1[i])/(s1[i]*s1[i]))-m1[i]

b1[i]<-a1[i]*(1-m1[i])/m1[i]

a2[i]<-m2[i]*m2[i]*((1-m2[i])/(s2[i]*s2[i]))-m2[i]

b2[i]<-a2[i]*(1-m2[i])/m2[i]

}

}

list(

T=1,x1=structure(.Data=c(36),.Dim=c(1,1)),

z=structure(.Data=c(34),.Dim=c(1,1)),zone=1,

m1=c(0.0923076923),s1=c(0.0359030708),

m2=c(0.0638297872),s2=c(0.0252130381),

Mv=c(74), mean_m=c(180.8181818182), Mvar=c(34.7847539164),

pmin=c(0.0351),pmax=c(0.357),

Mv2=c(55)

)

#Zone B

model {

for (i in 1:zone) {

for( t in 1 : T ) {

x1[i,t]~dbin(p[i,t],n1[i])

z[i,t]~dbin(p[i,t],n2[i])

x2[i,t]~dbin(p[i,t],n3[i])

p[i,t]~dunif(pmin[i],pmax[i])

}

n1[i]<-round(Mv[i])

n2[i]<-round((M[i]-Mv[i])+N[i])

n3[i]<-round(M[i]-Mv[i])

M[i]~dnorm(mean_m[i], Mtau[i])

Mtau[i]<-1/Mvar[i]

a[i]~dunif(0, 0.3)

N[i]<-a[i]*M[i]

vc1[i]<-Mv[i]/M[i]

vc2[i]<-Mv[i]/(M[i]+N[i])

vc1sec[i]<-(Mv[i]+Mv2[i])/M[i]

vc2sec[i]<-(Mv[i]+Mv2[i])/(M[i]+N[i])

}

}

list(

T=1,x1=structure(.Data=c(52),.Dim=c(1,1)),

z=structure(.Data=c(21),.Dim=c(1,1)),zone=1,

Mv=c(146), mean_m=c(304.7173913043), Mvar=c(21.3724857543),

pmin=c(0.0401),pmax=c(0.2558),

Mv2=c(89)

)

#Zone C

model {

for (i in 1:zone) {

for( t in 1 : T ) {

x1[i,t]~dbin(p[i,t],n1[i])

z[i,t]~dbin(p[i,t],n2[i])

x2[i,t]~dbin(p[i,t],n3[i])

p[i,t]~dunif(pmin[i],pmax[i])

}

n1[i]<-round((1-c1[i])*Mv[i])

n2[i]<-round((M[i]-Mv[i])+N[i])

n3[i]<-round(M[i]-Mv[i])

M[i]~dnorm(mean_m[i], Mtau[i])

Mtau[i]<-1/Mvar[i]

a[i]~dunif(0, 0.3)

N[i]<-a[i]*M[i]

c1[i]~dbeta(a1[i],b1[i])

vc1[i]<-Mv[i]/M[i]

vc2[i]<-Mv[i]/(M[i]+N[i])

vc1sec[i]<-(Mv[i]+Mv2[i])/M[i]

vc2sec[i]<-(Mv[i]+Mv2[i])/(M[i]+N[i])

a1[i]<-m1[i]*m1[i]*((1-m1[i])/(s1[i]*s1[i]))-m1[i]

b1[i]<-a1[i]*(1-m1[i])/m1[i]

}

}

list(

T=1,x1=structure(.Data=c(56),.Dim=c(1,1)),

z=structure(.Data=c(15),.Dim=c(1,1)),zone=1,

m1=c(0.0149253731),s1=c(0.0148135710),

Mv=c(74), mean_m=c(145.6911764706), Mvar=c(14.2251673687),

pmin=c(0.0307),pmax=c(0.4599),

Mv2=c(9)

)

#Zone D

model {

for (i in 1:zone) {

for( t in 1 : T ) {

x1[i,t]~dbin(p[i,t],n1[i])

z[i,t]~dbin(p[i,t],n2[i])

x2[i,t]~dbin(p[i,t],n3[i])

p[i,t]~dunif(pmin[i],pmax[i])

}

n1[i]<-round((1-c1[i])*Mv[i])

n2[i]<-round((M[i]-Mv[i])+N[i])

n3[i]<-round(M[i]-Mv[i])

M[i]~dnorm(mean_m[i], Mtau[i])

Mtau[i]<-1/Mvar[i]

a[i]~dunif(0, 0.3)

N[i]<-a[i]*M[i]

c1[i]~dbeta(a1[i],b1[i])

vc1[i]<-Mv[i]/M[i]

vc2[i]<-Mv[i]/(M[i]+N[i])

vc1sec[i]<-(Mv[i]+Mv2[i])/M[i]

vc2sec[i]<-(Mv[i]+Mv2[i])/(M[i]+N[i])

a1[i]<-m1[i]*m1[i]*((1-m1[i])/(s1[i]*s1[i]))-m1[i]

b1[i]<-a1[i]*(1-m1[i])/m1[i]

}

}

list(

T=1,x1=structure(.Data=c(21),.Dim=c(1,1)),

z=structure(.Data=c(37),.Dim=c(1,1)),zone=1,

m1=c(0.0169491525),s1=c(0.0168049018),

Mv=c(69), mean_m=c(338.6721311475), Mvar=c(189.9541399728),

pmin=c(0.0231),pmax=c(0.3079),

Mv2=c(122)

)
