# Supplementary material for: Domestic dog demographics and estimates of canine vaccination coverage in a rural area of Zambia for the elimination of rabies
Source: PLoS Negl Trop Dis. 2021 Apr 28;15(4):e0009222. doi: 10.1371/journal.pntd.0009222 (PMC8081203; doi:10.1371/journal.pntd.0009222)
Supplement: S4 Appendix — (DOCX) [file pntd.0009222.s004.docx]

**S4 Appendix. Formulas for calculating the life history parameters**

| *S*(*x*): Number of individuals sampled per age class *x*, which was smoothed by mean of equation 7 in the main text. |
| --- |
| *l*(*x*): Cumulative survival from the first age class *x1* to a given age class *x*.  $l(x)=\frac{s(x)}{s(x1)}$ |
| *p*(*x*): Probability of surviving from a given age class *x* to the next following age class (*x + 1*).  $p(x)=\frac{s(x+1)}{s(x)}$ |
| *d*(*x*): Overall mortality from the first age class *x1* to a given age class *x*.  $d\left( x \right)=1-l(x)$ |
| *q*(*x*): Age-specific mortality before reaching the next following age class.  $q\left( x \right)=1-p(x)$ |
| *e*(*x*): Age-specific life expectancy at a given age class *x*.  $e\left( x \right)=\frac{\sum_{x}^{xn} l\left( y \right)}{l\left( x \right)},$  *y* is summed from age class *x* to age class *xn* at the end of life. |
| *m*(*x*): Mean number of female pups born per age class  $m\left( x \right)=B\left( x \right)*b\left( x \right)*0.5$*,*  proportion of female pups was estimated at 0.5. |
