# Supplementary material for: Domestic dog demographics and estimates of canine vaccination coverage in a rural area of Zambia for the elimination of rabies
Source: PLoS Negl Trop Dis. 2021 Apr 28;15(4):e0009222. doi: 10.1371/journal.pntd.0009222 (PMC8081203; doi:10.1371/journal.pntd.0009222)
Supplement: S1 Table — (DOCX) [file pntd.0009222.s005.docx]

**S1 Table. Prior distributions**

|  | **Zone A** | **Zone B** | **Zone C** | **Zone D** |
| --- | --- | --- | --- | --- |
| Recapture *p_i_*^†^ |  |  |  |  |
| Uniform (range) | 0.035–0.357 | 0.040–0.256 | 0.031–0.460 | 0.023–0.308 |
| Coverage (*C_i_*) | 0.056–0.401 | 0.064–0.287 | 0.049–0.516 | 0.037–0.346 |
| Encountering (*E_i_*) | 0.70–0.90 | 0.70–0.90 | 0.70–0.90 | 0.70–0.90 |
| Recording (*R_i_*) | 0.90–0.99 | 0.90–0.99 | 0.90–0.99 | 0.90–0.99 |
| Confinement *c*_1,_*_i_* |  |  |  |  |
| Beta (*α*, *β*) |  |  |  |  |
| *α* | 5.908 | NIL^‡^ | 0.985 | 0.983 |
| *β* | 58.092 | NIL^‡^ | 65.015 | 57.017 |
| Confinement *c*_2,_*_i_* |  |  |  |  |
| Beta (*α*, *β*) |  |  |  |  |
| *α* | 5.936 | NIL^‡^ | NIL^‡^ | NIL^‡^ |
| *β* | 87.064 | NIL^‡^ | NIL^‡^ | NIL^‡^ |

† *pi* = *Ci***Ei***Ri*

‡ No confinement was observed in the zone
