# Supplementary material for: Domestic dog demographics and estimates of canine vaccination coverage in a rural area of Zambia for the elimination of rabies
Source: PLoS Negl Trop Dis. 2021 Apr 28;15(4):e0009222. doi: 10.1371/journal.pntd.0009222 (PMC8081203; doi:10.1371/journal.pntd.0009222)
Supplement: S2 Table — (DOCX) [file pntd.0009222.s006.docx]

**S2 Table. Overall population demographics (Static life table)**

| **Age class** | ***n* years** | **smoothed frequency *s*(*x*)** | **% *s*(*x*)** | ***l*(*x*)** | ***p*(*x*)** | ***d*(*x*)** | ***q*(*x*)** | ***e*(*x*)** |
| --- | --- | --- | --- | --- | --- | --- | --- | --- |
| 0–1 | 1 | 253 | 31.55 | 1.00 | 0.53 | 0.00 | 0.47 | 3.17 |
| 1–2 | 1 | 133 | 16.58 | 0.53 | 0.81 | 0.47 | 0.19 | 4.13 |
| 2–3 | 1 | 108 | 13.47 | 0.43 | 0.80 | 0.57 | 0.20 | 3.85 |
| 3–4 | 1 | 86 | 10.72 | 0.34 | 0.77 | 0.66 | 0.23 | 3.58 |
| 4–5 | 1 | 66 | 8.23 | 0.26 | 0.76 | 0.74 | 0.24 | 3.36 |
| 5–6 | 1 | 50 | 6.23 | 0.20 | 0.74 | 0.80 | 0.26 | 3.12 |
| 6–7 | 1 | 37 | 4.61 | 0.15 | 0.70 | 0.85 | 0.30 | 2.86 |
| 7–8 | 1 | 26 | 3.24 | 0.10 | 0.69 | 0.90 | 0.31 | 2.65 |
| 8–9 | 1 | 18 | 2.24 | 0.07 | 0.67 | 0.93 | 0.33 | 2.39 |
| 9–10 | 1 | 12 | 1.50 | 0.05 | 0.67 | 0.95 | 0.33 | 2.08 |
| 10–11 | 1 | 8 | 1.00 | 0.03 | 0.63 | 0.97 | 0.38 | 1.63 |
| 11+ | 12 | 5 | 0.62 | 0.02 | 0.00 | 0.98 | 1.00 | 1.00 |

Age class: age in years

n year: number of years spent in the age class

*s*(*x*): smoothed number of individuals sampled per age class

*s*(*x*) %: percentage of sample per age class

*l*(*x*): cumulative survival

*p*(*x*): age-specific survival from age *x* to age *x*+1

*d*(*x*): cumulative mortality

*q*(*x*): age-specific mortality from age *x* to age *x*+1

*e*(*x*): age-specific life expectancy
