# Supplementary material for: Domestic dog demographics and estimates of canine vaccination coverage in a rural area of Zambia for the elimination of rabies
Source: PLoS Negl Trop Dis. 2021 Apr 28;15(4):e0009222. doi: 10.1371/journal.pntd.0009222 (PMC8081203; doi:10.1371/journal.pntd.0009222)
Supplement: S3 Table — (DOCX) [file pntd.0009222.s007.docx]

**S3 Table. Female fecundity**

| **Age class** | **smoothed frequency *s*(*x*)** | ***b*(*x*)** | ***B*(*x*)** | ***m*(*x*)** |
| --- | --- | --- | --- | --- |
| 0–1 | 114 | 0.00 | 0.00 | 0.00 |
| 1–2 | 64 | 0.34 | 4.08 | 0.70 |
| 2–3 | 44 | 0.58 | 4.03 | 1.17 |
| 3–4 | 30 | 0.53 | 4.27 | 1.14 |
| 4–5 | 21 | 0.76 | 4.59 | 1.74 |
| 5–6 | 14 | 0.81 | 4.67 | 1.89 |
| 6–7 | 10 | 0.40 | 3.60 | 0.72 |
| 7–8 | 7 | 1.00 | 3.50 | 1.75 |
| 8–9 | 5 | 0.83 | 4.33 | 1.81 |
| 9–10 | 3 | 0.50 | 4.00 | 1.00 |
| 10–11 | 2 | 0.75 | 4.00 | 1.50 |
| 11+ | 2 | 0.50 | 2.50 | 0.63 |

Age class: age in years

*s*(*x*): smoothed number of individuals sampled per age class

*b*(*x*): mean proportion of breeding females per year and age class

*B*(*x*): mean number of offspring born in the last litter per female and age class

*m*(*x*): number of female pups born per female and year
