# Supplementary material for: Domestic dog demographics and estimates of canine vaccination coverage in a rural area of Zambia for the elimination of rabies
Source: PLoS Negl Trop Dis. 2021 Apr 28;15(4):e0009222. doi: 10.1371/journal.pntd.0009222 (PMC8081203; doi:10.1371/journal.pntd.0009222)
Supplement: S4 Table — (DOCX) [file pntd.0009222.s008.docx]

**S4 Table. Sex-specific population demographics (Static life tables)**

| **a. Static life table of male dogs** | | | | | | | | | |
| --- | --- | --- | --- | --- | --- | --- | --- | --- | --- |
| **Age class** | ***n* years** | **smoothed frequency *s*(*x*)** | **% *s*(*x*)** | ***l*(*x*)** | ***p*(*x*)** | ***d*(*x*)** | ***q*(*x*)** | ***e*(*x*)** |  |
| 0–1 | 1 | 120 | 27.76 | 1.00 | 0.54 | 0.00 | 0.46 | 3.60 |  |
| 1–2 | 1 | 65 | 15.01 | 0.54 | 0.88 | 0.46 | 0.12 | 4.81 |  |
| 2–3 | 1 | 57 | 13.18 | 0.47 | 0.85 | 0.53 | 0.15 | 4.34 |  |
| 3–4 | 1 | 48 | 11.17 | 0.40 | 0.82 | 0.60 | 0.18 | 3.94 |  |
| 4–5 | 1 | 39 | 9.14 | 0.33 | 0.79 | 0.67 | 0.21 | 3.60 |  |
| 5–6 | 1 | 31 | 7.22 | 0.26 | 0.76 | 0.74 | 0.24 | 3.29 |  |
| 6–7 | 1 | 24 | 5.50 | 0.20 | 0.74 | 0.80 | 0.26 | 3.01 |  |
| 7–8 | 1 | 17 | 4.05 | 0.15 | 0.71 | 0.85 | 0.29 | 2.73 |  |
| 8–9 | 1 | 12 | 2.87 | 0.10 | 0.69 | 0.90 | 0.31 | 2.43 |  |
| 9–10 | 1 | 9 | 1.97 | 0.07 | 0.66 | 0.93 | 0.34 | 2.08 |  |
| 10–11 | 1 | 6 | 1.30 | 0.05 | 0.64 | 0.95 | 0.36 | 1.64 |  |
| 11+ | 12 | 4 | 0.83 | 0.03 | 0.00 | 0.97 | 1.00 | 1.00 |  |
|  | | | | | | | | | |
| **b. Static life table of female dogs** | | | | | | | | |  |
| **Age class** | ***n* years** | **smoothed frequency *s*(*x*)** | **% *s*(*x*)** | ***l*(*x*)** | ***p*(*x*)** | ***d*(*x*)** | ***q*(*x*)** | ***e*(*x*)** |  |
| 0–1 | 1 | 118 | 33.57 | 1.00 | 0.59 | 0.00 | 0.41 | 2.98 |  |
| 1–2 | 1 | 69 | 19.70 | 0.59 | 0.73 | 0.41 | 0.27 | 3.37 |  |
| 2–3 | 1 | 50 | 14.30 | 0.43 | 0.72 | 0.57 | 0.28 | 3.27 |  |
| 3–4 | 1 | 36 | 10.26 | 0.31 | 0.71 | 0.69 | 0.29 | 3.16 |  |
| 4–5 | 1 | 26 | 7.27 | 0.22 | 0.70 | 0.78 | 0.30 | 3.05 |  |
| 5–6 | 1 | 18 | 5.09 | 0.15 | 0.69 | 0.85 | 0.31 | 2.93 |  |
| 6–7 | 1 | 12 | 3.52 | 0.10 | 0.68 | 0.90 | 0.32 | 2.79 |  |
| 7–8 | 1 | 8 | 2.40 | 0.07 | 0.67 | 0.93 | 0.33 | 2.61 |  |
| 8–9 | 1 | 6 | 1.62 | 0.05 | 0.67 | 0.95 | 0.33 | 2.39 |  |
| 9–10 | 1 | 4 | 1.08 | 0.03 | 0.66 | 0.97 | 0.34 | 2.09 |  |
| 10–11 | 1 | 3 | 0.71 | 0.02 | 0.65 | 0.98 | 0.35 | 1.65 |  |
| 11+ | 5 | 2 | 0.46 | 0.01 | 0.00 | 0.99 | 1.00 | 1.00 |  |

Age class: age in year

n year: number of years spent in the age class

*s*(*x*): number of individuals sampled per age class

*s*(*x*) %: percentage of sample per age class

*l*(*x*): cumulative survival

*p*(*x*): age-specific survival from age *x* to age *x*+1

*d*(*x*): cumulative mortality

*q*(*x*): age-specific mortality from age *x* to age *x*+1

*e*(*x*): age-specific life expectancy
