# Supplementary material for: Domestic dog demographics and estimates of canine vaccination coverage in a rural area of Zambia for the elimination of rabies
Source: PLoS Negl Trop Dis. 2021 Apr 28;15(4):e0009222. doi: 10.1371/journal.pntd.0009222 (PMC8081203; doi:10.1371/journal.pntd.0009222)
Supplement: S5 Table — (DOCX) [file pntd.0009222.s009.docx]

**S5 Table. Posterior distributions**

|  | **Zone A** | **Zone B** | **Zone C** | **Zone D** |
| --- | --- | --- | --- | --- |
| Estimated owned dog population (*M_i_*) | | | | |
|  | 179 | 302 | 140 | 329 |
|  | (168–190) | (292–311) | (133–148) | (302–357) |
| Ratio of ownerless to owned dogs (*a_i_*) | | | | |
|  | 0.06 | 0.02 | 0.01 | 0.05 |
|  | (0.00–0.23) | (0.00–0.10) | (0.00–0.08) | (0.00–0.23) |
| Probability to recapture dogs (*p_i_*) | | | | |
|  | 0.34 | 0.23 | 0.44 | 0.17 |
|  | (0.29–0.36) | (0.19–0.25) | (0.39–0.46) | (0.13–0.22) |
| Confinement probability for owned marked dogs (*c*_1_*_,i_*) | | | | |
|  | 0.07 | NIL^‡^ | 0.00 | 0.01 |
|  | (0.03–0.13) |  | (0.00–0.03) | (0.00–0.05) |
| Confinement probability for owned unmarked dogs (*c*_2_*_,i_*) | | | | |
|  | 0.06 | NIL^‡^ | NIL^‡^ | NIL^‡^ |
|  | (0.03–0.13) |  |  |  |

‡ No confinement was observed in the zone

Values in parentheses are 95 % credible intervals

The number of digits after the decimal point was truncated in the number of dogs.
