# Supplementary material for: Domestic dog demographics and estimates of canine vaccination coverage in a rural area of Zambia for the elimination of rabies
Source: PLoS Negl Trop Dis. 2021 Apr 28;15(4):e0009222. doi: 10.1371/journal.pntd.0009222 (PMC8081203; doi:10.1371/journal.pntd.0009222)
Supplement: S7 Table — (DOCX) [file pntd.0009222.s011.docx]

**S7 Table. Proportion of vaccinated owned dogs based on the observation in the household survey**

|  | **Zone A** | **Zone B** | **Zone C** | **Zone D** |
| --- | --- | --- | --- | --- |
| Number of owned dogs observed in the household survey | 159 | 286 | 132 | 295 |
| Proportion of vaccinated owned dogs through the first mass vaccination campaign (%)^†^ | 40.9 | 47.9 | 50.8 | 20.3 |
| Proportion of vaccinated owned dogs through the first and follow-up mass vaccination campaign (%)^†,‡^ | 75.5 | 79.0 | 57.6 | 61.7 |

† The number of vaccinated dogs in the first mass vaccination is based on the number of marked dogs recaptured in the household survey.

‡ The number of vaccinated dogs in the follow-up vaccination is based on the number of registration in the follow-up mass vaccination sites.
