# Supplementary material for: Domestic dog demographics and estimates of canine vaccination coverage in a rural area of Zambia for the elimination of rabies
Source: PLoS Negl Trop Dis. 2021 Apr 28;15(4):e0009222. doi: 10.1371/journal.pntd.0009222 (PMC8081203; doi:10.1371/journal.pntd.0009222)
Supplement: S8 Table — (DOCX) [file pntd.0009222.s012.docx]

| **Reason** | **Answers (*n*)** | **%** |
| --- | --- | --- |
| Not informed | 58 | 32.0 |
| Owner’s unavailability (Owner went to church/work/funeral/hospital etc.) | 48 | 26.5 |
| Owner failed to restrain his/her dog(s) | 43 | 23.8 |
| Mistime/misunderstood the venue | 9 | 5.0 |
| Vaccine was still valid | 5 | 2.8 |
| Dog was too young for vaccination | 4 | 2.2 |
| Owner was reluctant | 2 | 1.1 |
| Owner was sick | 2 | 1.1 |
| Owner misunderstood that his/her dogs had valid certificates | 1 | 0.6 |
| Owner doubted if it was free vaccination or not | 1 | 0.6 |
| Owner’s house was far from the vaccine site | 1 | 0.6 |
| Owner had not yet got dog at the time of the vaccination | 1 | 0.6 |
| Unavailable answers | 6 | 3.3 |
| Total number of respondents | 181 | 100 |

**S8 Table. Reasons for non-participation in the first mass vaccination campaign**
